# Analysis of Human Mutations in the Supernumerary Subunits of Complex I

**DOI:** 10.3390/life10110296

**Published:** 2020-11-20

**Authors:** Quynh-Chi L. Dang, Duong H. Phan, Abigail N. Johnson, Mukund Pasapuleti, Hind A. Alkhaldi, Fang Zhang, Steven B. Vik

**Affiliations:** Department of Biological Sciences, Southern Methodist University, Dallas, TX 75287, USA; quynhchid@smu.edu (Q.-C.L.D.); lisap@smu.edu (D.H.P.); abbyjohnson@smu.edu (A.N.J.); mpasapuleti@smu.edu (M.P.); halkhaldi@smu.edu (H.A.A.); fangz@smu.edu (F.Z.)

**Keywords:** mitochondria, mammalian complex I, NADH dehydrogenase, complex I assembly, complex I structure, complex I deficiency, supernumerary subunits, electron transport chain, mitochondrial dysfunction, Leigh syndrome

## Abstract

Complex I is the largest member of the electron transport chain in human mitochondria. It comprises 45 subunits and requires at least 15 assembly factors. The subunits can be divided into 14 “core” subunits that carry out oxidation–reduction reactions and proton translocation, as well as 31 additional supernumerary (or accessory) subunits whose functions are less well known. Diminished levels of complex I activity are seen in many mitochondrial disease states. This review seeks to tabulate mutations in the supernumerary subunits of humans that appear to cause disease. Mutations in 20 of the supernumerary subunits have been identified. The mutations were analyzed in light of the tertiary and quaternary structure of human complex I (PDB id = 5xtd). Mutations were found that might disrupt the folding of that subunit or that would weaken binding to another subunit. In some cases, it appeared that no protein was made or, at least, could not be detected. A very common outcome is the lack of assembly of complex I when supernumerary subunits are mutated or missing. We suggest that poor assembly is the result of disrupting the large network of subunit interactions that the supernumerary subunits typically engage in.

## 1. Introduction

Mutations in the genes that encode complex I are responsible for a large fraction of all mitochondrial diseases. For example, 20–30% of cases in childhood mitochondrial disease (MD) are related to complex I dysfunction [1,2]. There seem to be many reasons for this. Complex I genes make up a large component of the mitochondrial and nuclear genome. Complex I is encoded by seven mitochondrial genes (out of 13 total) and 37 nuclear genes. There are also at least 15 complex I assembly factors [3]. Mitochondrial DNA (mtDNA) is especially prone to mutation due to insufficient DNA repair systems. All of the various functions of complex I can be impacted by mutation. Complex I forms supercomplexes with other members of the respiratory chain, such as complex III (cytochrome *bc_1_*) and complex IV (cytochrome *c* oxidase). It is metabolically linked to the citric acid cycle by NADH and to ATP synthesis by proton translocation. Furthermore, it is a major site of superoxide generation in mitochondria. Therefore, mutations that alter or degrade complex I function will typically have wider effects on mitochondrial function.

**Figure 1:**
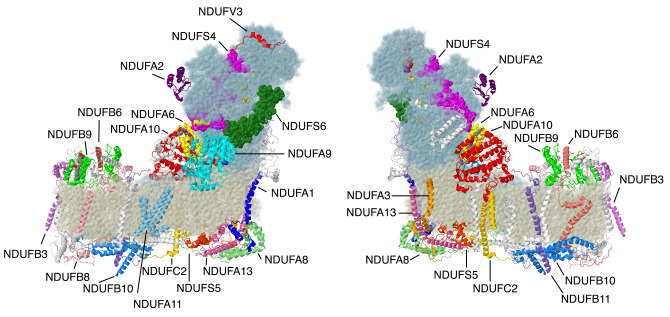
Structure of complex I with supernumerary subunits highlighted. Core subunits in the matrix arm are colored light blue. Core subunits in the membrane arm are colored beige. Supernumerary subunits that are described in this review are colored and labeled. Other supernumerary subunits are white. The two views are rotated 180° relative to each other. The structure is from the Protein Data Bank file 5xtd (PDB id = 5xtd) [9]. All structural images were generated using Jmol (http://www.jmol.org).

Complex I is a boot-shaped multi-subunit enzyme embedded in the inner mitochondrial membrane (see Figure 1). Its primary role is to oxidize NADH while reducing ubiquinone and translocating protons across the membrane. One arm extends into the matrix space, and it contains the flavin mononucleotide (FMN) and all of the iron–sulfur (FeS) clusters necessary for electron transfer to ubiquinone. The membrane arm contains subunits that translocate the protons from the matrix space to the intermembrane space (IMS). These core functions are carried out by the fourteen “core” subunits that appear in all known examples of complex I, including bacteria. Seven of the core subunits are membrane-embedded, and these seven are all encoded in mtDNA: ND1, ND2, ND3, ND4, ND4L, ND5, and ND6. The other seven core subunits are found in the matrix arm and contain the FMN and all of the FeS clusters. NDUFV1 contains the flavin and one tetranuclear FeS cluster, N3. NDUFV2 contains one binuclear FeS cluster N1a, which is not on the main electron transfer path. NDUFS1 contains one binuclear FeS cluster, N1b, and two tetranuclear clusters, N4 and N5. NDUFS8 contains two ferredoxin-like clusters, N6a and N6b. NDUFS7 contains the tetranuclear cluster N2, which is proximal to the ubiquinone binding site. For recent reviews of complex I, see [4,5,6,7]; for supernumerary subunits, see [8].

The remaining thirty-one subunits (one is found in two copies) are supernumerary (or accessory) subunits, and much less is known about their functions. They are typically much smaller than the core subunits, and they are distributed on all surfaces of complex I. Some cross the membrane, while others are localized to the matrix face or the IMS. The naming of these subunits has generally followed their co-purification with various fractions of complex I: FV for the flavoprotein fraction, FS for the FeS protein fraction, FA for the alpha fraction associated with the matrix arm subunits, and FB for the beta fraction associated with the membrane proteins. The exception is NDUFAB1, the acyl carrier protein, which resembles an enzyme in lipid biosynthesis. This subunit appears to have an essential role apart from complex I, and it is the only protein that appears in two copies.

From an evolutionary point of view, the core subunits can be organized into three modules. The N-module contains NDUFV1, NDUFV2, and NDUFS1, and it is defined by the source of electrons to the complex, the substrate NADH. This module is related to various NAD-linked dehydrogenases. The Q-module contains the remaining peripheral subunits NDUFS2, NDUFS3, NDUFS7, and NDUFS8, as well as two membrane subunits, ND1 and ND3, that contain the remaining FeS clusters and contribute to the ubiquinone binding site. This module is related to various membrane-bound hydrogenases. Finally, the remaining membrane subunits, ND2, ND4, ND4L, ND5, and ND6 compose the P-module (for proton translocating) and are related to subunits of an Na^+^/H^+^ antiporter, the Mrp complex [10]. This grouping of subunits also corresponds to the assembly pathway. Q-module subunits appear to assemble first, followed by the stepwise addition of the P-module, associated with the three major subunits ND2, ND4, and ND5. ND5 and the N-module enter the complex last.

In this review, 20 supernumerary subunits for which clinical mutations have been identified are described (see Table 1). Some of the mutations are interpreted in light of the structure of the human complex I (Protein Data Bank file 5xtd) or, in some cases, from other species. Some of the mutations are likely to be null mutations in which no protein is made, but the evidence is not always clear for that. Currently, the understanding of the effects of mutations is limited by a lack of knowledge. For example, how do mutations in one subunit affect the stability or import of that subunit? How does the absence of one subunit affect the expression, import, or stability of other subunits? How do mutations affect the assembly of that subunit or of other subunits? Do supernumerary subunits have roles that can be affected by mutation while assembly remains normal?

## 2. Review of Mutations in the Supernumerary Subunits

### 2.1. N-Module Subunits

**Figure 2:**
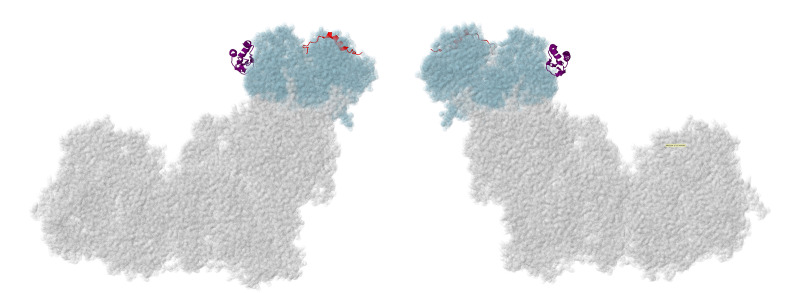
Location of NDUFA2 and NDUFV3. Most of Complex I is colored gray. Core subunits in the N-module arm are colored light blue (NDUFV1, NDUFV2, and NDUFS1). NDUFA2 and NDUFV3 are shown in ribbons, with NDUFA2 colored purple and NDUFV3 colored red. The two views are rotated 180° relative to each other. The structure is from PDB id = 5xtd [9].

The first subunits to be described are two subunits that primarily have contacts to core subunits of the N-module (see Supplementary File Table S1 for a Table of Subunit Interactions). Overall, they are rather isolated from other supernumerary subunits, as shown in Figure 2. Beyond those similarities, their properties appear divergent.

#### 2.1.1. NDUFA2

**Figure 3:**
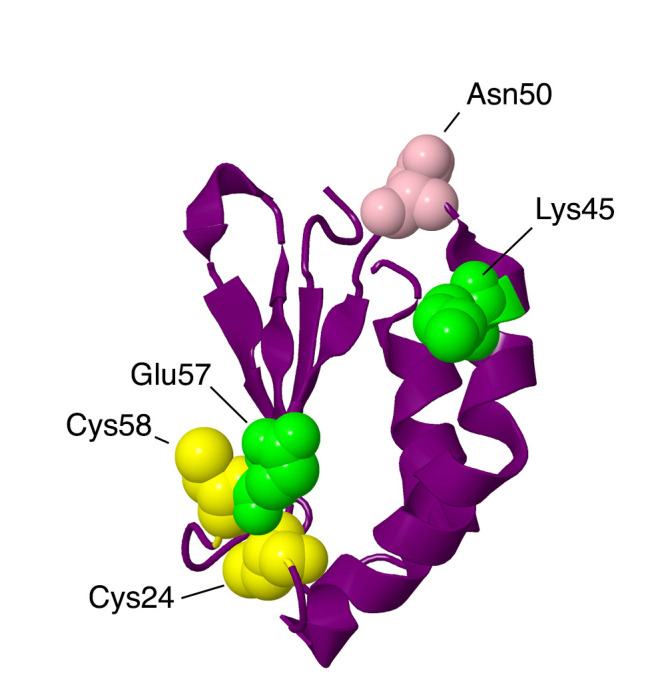
Structural features of NDUFA2 from the N-module. The protein is portrayed in a purple-colored ribbon. Sites of mutations are shown in space-filling views: Lys45Thr and Glu57Ala are mitochondrial disease mutations (colored lime). Asn50Asp has been found in breast cancer patients (colored pink). Two Cys residues are colored yellow.

The gene for NDUFA2 is located on chromosome 5. The protein contains 99 amino acids with a mass of about 10.9 kDa before the processing of the amino-terminal methionine. This subunit is bound to the matrix arm in a unique fashion, with exclusive contacts to core subunit NDUFS1. It is bound to C-terminal domains of NDUFS1, and it is at least 40 Å from any of the FeS clusters. It has a compact structure that resembles a thioredoxin fold and includes a flat four-stranded beta-sheet (see Figure 3). Two Cys residues, 24 and 58, are found in the loops of the beta-sheet, but they do not appear to be oxidized in the existing structures of mammalian complex I. Contact with NDUFS1 is primarily with the beta-sheet region, including both Cys residues. In culture, NDUFA2 knockout cell lines have been found to lack complex I activity, and an analysis by blue native (BN) gel electrophoresis showed a limited assembly of the N-module—in particular, the loss of core subunits NDUFS1, NDUFV1, and NDUFV2 and supernumerary subunits NDUFS4, NDUFS6, NDUFA7, and NDUFV3. In a cell line with an NDUFA2 knockout, complex I assembly is slightly affected, with a prominent band in BN gel electrophoresis but apparently missing some subunits, probably the N-module. Complex I activity has been found to be extremely low, consistent with a missing NADH binding site [11].

An individual with Leigh syndrome and hypertrophic cardiomyopathy was discovered to be homozygous with a mutation, c.208 + 5 G > A, in the NDUFA2 gene that caused a reduction in correct splicing [12]. Normally, NDUFA2 is coded by three exons, but in this individual, most of the transcripts lacked exon 2, causing a frameshift. No protein or transcript was found, making it a null mutation. The patient died of cardiovascular arrest at eleven months.

Two individuals with leukoencephalopathy were discovered to have mutations in NDUFA2 [13]. One was homozygous for p.Lys45Thr, and the other had compound heterozygous mutations, p.Lys45Thr and a deletion at Asn76, c.225del, and p.Asn76Metfs*4, thus causing a frameshift and a stop codon after 4 codons. The first was found to have complex I deficiency and lacked an assembled complex I in BN gel electrophoresis. This individual also had a systemic deficiency in carnitine due to a homozygous mutation in SLC22A5. She developed focal epilepsy at six years, was wheelchair-bound at nine years, and was last evaluated at 12 years. The second patient had a similar presentation, including abnormal white matter in the brain. She also had movement difficulties and was last evaluated at age four. Lys45 is found at the interface with NDUFS1 and has close contacts with several NDUFS1 residues, including Gly376, Asp380, and Ser672. It is likely that the binding of NDUFA2 to NDUFS1 is destabilized.

In 2020 [14], another individual was described with a homozygous mutation in NDUFA2, p.Glu57Ala. This residue is at the interface with NDUFS1 and makes a possible ion pair with Arg382 of NDUFS1. It also makes nonbonding contacts with Gly661, Ala662, Asn663, Tyr664, Leu381, Arg382, and Ser383, suggesting a likely disruption of binding. This individual had abnormal white matter in the brain, microcephaly, seizures, and movement disorders. She was last evaluated at four years of age.

In a screen of breast cancer patients [15], a homozygous p.Asp50Asn mutation in NDUFA2 was detected in one individual. This residue is not at the interface with NDUFS1, and so it perhaps does not disrupt the assembly of the enzyme. The significance of this finding is uncertain.

#### 2.1.2. NDUFV3

The gene for NDUFV3 is found on chromosome 21. This was the first subunit of human complex I known to have multiple isoforms [16,17,18], which can be found by alternative splicing. It is not an essential protein, as shown by knockout studies in cell culture [11]. The loss of this gene does not prevent the assembly of complex I, as seen in BN gels, and cells retain the ability to grow in a galactose medium, indicative of oxidative phosphorylation activity. Exogenous NDUFV3 has been seen to exchange with fully assembled complex I, suggesting that it can be incorporated last into the enzyme. The more common isoform has 108 amino acids (74 after the cleavage of the transit peptide) and a mass of 11.8 kDa, while the second isoform has 473 amino acids and a mass of about 51 kDa. Isoform two has an additional exon between the second and the last exons of the shorter form. The additional exon contains a serine-rich region that was found to be phosphorylated [19]. NDUFV3 is not present in fungal complex I from *Yarrowia lipolytica* [7].

NDUFV3 was originally identified in the flavoprotein fraction of bovine heart complex I and now can be seen to have primary contacts with NDUFV1 and NDUFV2 in the human enzyme. It also has limited contact with NDUFS1 and NDUFS4. In the human model of complex I (PDB id = 5xtd) [9], only 33 amino acids are visible, corresponding to residues 74–106. Residues 82–5 are alpha-helical. The longer isoform has an insertion at residue 56 and retains the same C-terminus as isoform one. Therefore, both isoforms can likely bind in the same fashion to other complex I subunits. Both isoforms have been seen in a variety of tissues, but isoform two is more common in cultured cells and in brain tissue [16]. Others have found that isoform one is more prevalent in bovine and murine complex I of heart tissue, as well as that increased Km values for NADH consumption by complex I correlate with an increased extent of the short form in heart tissue [18]. In a cross-linking study of native mouse heart mitochondria [20], both isoforms were identified in cross-links with a surface peptide of malate dehydrogenase, found near the NAD^+^ binding site. The binding of malate dehydrogenase and several other enzymes to porcine complex I was demonstrated in 1984 [21]. Other cross-links identified with NDUFV3 were with NDUFV1, NDUFV2, Cox7A1, and Atp5a1—the latter two supporting the proximity of the complexes of oxidative phosphorylation.

Sequence variations have been discovered in NDUFV3 among individuals with decreased levels of complex I activity, but none have been shown to be causative for disease. In one such cohort, four individuals with mutations in NDUFV3 were found [22]. The first individual had p.Arg26Gln, which would occur in the mitochondrial transit peptide and thus might affect import. A second mutation, was found in the same individual in the mitochondrial DNA polymerase G gene POLG, p.Gly11Asp. The second individual had the mutation p.Lys56Asn, which occurs at the splice junction and might affect expression, but this individual also carried a mutation in AMACR, an enzyme associated with branched-chain fatty acid metabolism, p.Val185Ala. The third individual had the mutation p.Gly103Asp, which would occur in both isoforms, in the region that binds to other subunits. Gly103 contacts Trp166 of NDUFS4 in a non-bonding interaction, and it is adjacent to several charged residues. Arg104 of NDUFV3 is near Arg169 of NDUFS4 and Asp426 of core subunit NDUFS1. Glu105 of NDUFV3 and Glu72 of NDUFV2 are nearby. Ser106 is also in this region, and it was found to be phosphorylated in a screen of human cancer cells [23]. Therefore the p.Gly103Asp mutation appears likely to be disruptive to the binding of NDUFV3. The fourth individual had the mutation p.Glu276Lys, which is found only in the long form, near the sites of phosphorylation. This individual also carried a p.Gly154Ser mutation in the core subunit NDUFS8, which is a buried residue, and that substitution is more likely to be deleterious.

### 2.2. Q-Module Subunits

**Figure 4:**
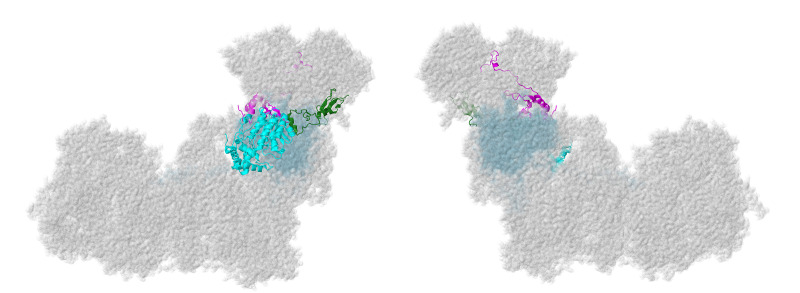
Location of NDUFS4, NDUFS6, and NDUFA9. Most of Complex I is colored gray. Core subunits in the Q-module are colored light blue (NDUFS2, NDUFS3, NDUFS7, and NDUFS8). NDUFS4, NDUFS6, and NDUFA9 are shown in ribbons, with NDUFS4 colored magenta, NDUFS6 colored green, and NDUFA9 colored cyan. The two views are rotated 180° relative to each other. The structure is from PDB id = 5xtd [9].

NDUFS4, NDUFS6, and NDUFA9 are found in the Q-module, as shown in Figure 4. They interact with each other and with many other subunits in the matrix arm. They appear to have roles in assembling and stabilizing the N- and Q-modules.

#### 2.2.1. NDUFS4

**Figure 5:**
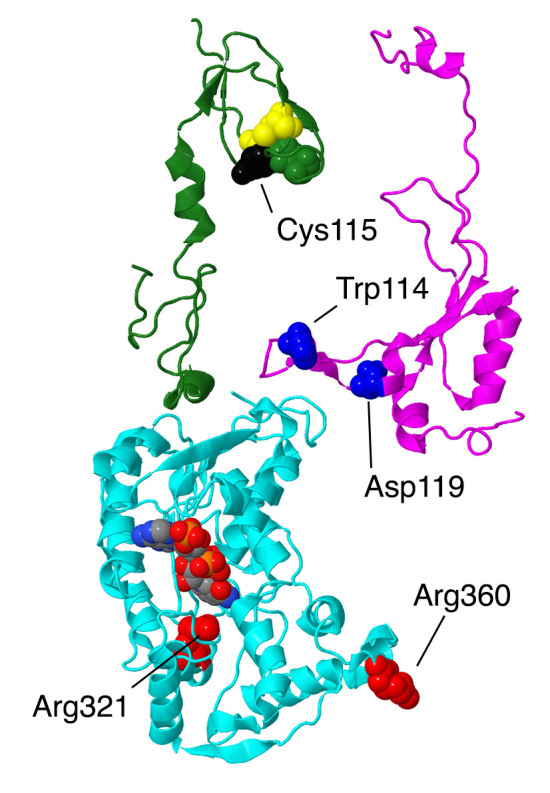
Structural features of 3 interacting subunits from the Q-module. The proteins are portrayed in ribbons. NDUFS4 is colored magenta. The sites of two mutations in this subunit, Trp114Arg and Asp119His, are shown in space-filling and are colored blue. NDUFS6 is colored green. The zinc bind residues are shown in space-filling, with Cys colored yellow and His colored green. The site of the mutation, Cys115Tyr is colored black. NDUFA9 is colored cyan, and its bound ligand NADP^+^ is shown in space-filling with CPK colors (e.g., carbon gray, nitrogen blue, oxygen red, phosphorus orange). The sites of 2 mutations, Arg321Pro and Arg360Cys, are shown in space-filling and are colored red.

The gene for NDUFS4 is found on chromosome 5. NDUFS4 is a protein that was initially found in the iron–sulfur protein fraction of bovine heart complex I. It is synthesized as 175 amino acids, and after the cleavage of the mitochondrial transit peptide, the mature protein is 133 amino acids with a mass of about 15.3 kDa. As shown in Figure 1, it is located in the matrix arm of complex I and contacts core subunits NDUFS1, NDUFS3, NDUFS8, and NDUFV1, as well as supernumerary subunits NDUFA6 and NDUFA9. It fits between two lobes of core subunit NDUFS1. It can be understood as a globular protein with a mixed alpha/beta architecture and three extensions that reach towards the chain of FeS clusters (see Figure 5). The C-terminus, in particular, is within about 9 Å of FeS clusters N3 and N1b, found in NDUFV1 and NDUFS1, respectively. A loop ending in Met87 is within 10 Å of FeS clusters N4 and N5 of NDUFS1, and a second loop including Met112 is pointing toward the last three FeS clusters—N6a, N6b, and N2—but is at least 13 Å away. NDUFS4 has been found to be phosphorylated at residue Ser173, near the C-terminus [24]. It has been suggested that this improves the net import of the subunit by inhibiting its return to the cytoplasm. In a knockout cell line of cultured human cells, the loss of NDUFS4 had a mild effect on assembly of complex I, causing it to migrate as a smaller-than-normal-sized complex in BN gel electrophoresis, probably lacking the N-module [11].

Three human mutations in NDUFS4 from Leigh syndrome patients were examined biochemically [25]: a duplication of AAGTC at position 466–470 of the coding sequence, a single G deletion at position 289 (in Trp97), and a nonsense mutation, c.44 G > A, p.Trp15X, in the first exon of the gene. Nonsense-mediated decay apparently eliminated the transcript in the second case. In the third case, c.44 G > A, it was later determined that three alternative splice variants were produced [26]. In all three cases, the mutations were homozygous, and it was demonstrated that little or no protein was made. An analysis of these null mutants from cultured fibroblasts showed that complex I did not assemble by BN gel electrophoresis and that little or no activity could be measured [25].

Two clinical missense mutations have been identified in this subunit, p.Trp114Arg [27] and p.Asp119His [28], both resulting in Leigh syndrome. Several frameshift mutations have been identified, including the homozygous c.221delC (p.Thr74Ifs*17) and the compound heterozygous mutations c.462delA (p.Lys154Nfs*34) and c.99-1 G > A) (p.Ser34Ifs*4), which also appear to cause Leigh syndrome [22]. Both missense mutations are found in the loop nearest to the N2/N6ab FeS clusters. Perhaps more importantly, this extension of NDFS4 is part of a junction of three subunits, with NDUFA9, the NADP^+^ binding protein, and an extension of core subunit NDUFS3. The individual with the p.Trp114Arg mutation was found to be homozygous for this allele, while both parents were found to be heterozygous [27]. This individual was diagnosed with myocarditis, respiratory failure, delirium, and basal ganglia abnormalities in the brain as seen by magnetic resonance imaging (MRI), and she was found to have complex I deficiency. Trp114 is a conserved residue that has close contact with Gln228 of NDUFS3 and is near several residues of core subunit NDUFS8. In the human structure (PDB id = 5xtd), the glutamine oxygen from the sidechain is pointing into the tryptophan ring, but it seems that the amino group would more logically assume that position. In conclusion, it seems likely that the p.Trp114Arg mutation would be disruptive and might impact the transfer of electrons through complex I.

The individual with the p.Asp119His mutation was compound heterozygous, with a second allele containing a Lys154 frame shift mutation, and he died in his third year [28]. While these mutations were found in a heterozygous situation (mother, father, and brother), there was no clinical presentation, suggesting that both mutations contributed to declining health. In a different study, the transcript for the frameshift allele was shown to exist, indicating that nonsense-mediated decay did not occur and suggesting that the protein was made. Asp119 is primarily contacted by other residues in NDUFS4, including an ion pairing with Arg75. The closest contact with subunit NDUFA9 is residue Lys45 at a distance of over 5 Å. Therefore, it is likely that the Asp119His substitution would be deleterious. If we assume that the frameshift mutation would produce a protein that is truncated at residue 154, that would remove a small but highly conserved domain that normally interacts with NDUFV3, and with core subunits NDUFV1, NDUFV2, and NDUFS1, in the vicinity of the FeS clusters N3 and N1b. Even this loss of about 20 amino acids would appear to be disruptive, and it seems possible that the truncated protein might be found at lower-than-normal levels due to instability.

#### 2.2.2. NDUFS6

The gene for NDUFS6 is located in chromosome 5 and contains four exons. The protein has 124 amino acids before the N-terminal 28 are cleaved upon entry to the mitochondrion. The original mass is 13.7 kDa. The protein is bound to the matrix arm and has two domains connected by a short alpha-helix, residues 67–75 (see Figure 5). The N-terminal domain, residues 29–66, is closer to the membrane and primarily contacts subunits NDUFA9 and NDUFA12. The C-terminal domain, residues 76–124, contains several short beta-strands and is in contact with core subunits NDUFS1, NDUFV2, NDUFS2, and NDUFS8. A zinc ion is bound by three cysteines and one histidine that are part of a C–X_8_–H–X_7_–C–X_2_–C motif with Cys87, Cys112, Cys115, and His96. The significance of the bound zinc is not clear, but this subunit is found not only in the fungal *Y. lipolytica* enzyme [29,30] but also in some bacterial enzymes (e.g., *Paracoccus denitrificans*) [31]. In cell culture, the knockout of NDUFS6 results in a mild assembly defect in which complexes of somewhat smaller size with a reduced oxidative phosphorylation capacity are seen [11]. In *Y. lipolytica*, the knockout of the NDUFS6 gene results in a complex lacking NDUFA12 and the N4 FeS cluster. Complementation with constructs lacking one of the zinc binding residues failed to regenerate the wild-type activity, suggesting a role in the incorporation of this FeS cluster [29].

The first mutations associated with NDUFS6 were described in 2004 [32]. Three individuals presented with lethal neonatal mitochondrial complex I deficiency and died within days of birth. Two were siblings with homozygous mutations, c.186 + 2 T > A, which affected splicing, leading to 26 bp of intronic RNA incorporated at the exon 2/3 junction, causing a frameshift and a protein predicted to be 71 amino acids. A small amount (3%) of normally spliced transcript was also found. The third individual was also homozygous, with a deletion of 4.75 kb that removed exons 3 and 4. This individual died five days after birth.

A mutation in one of the residues that bind zinc, p.Cys115Tyr, was found in two different families [33]. The affected individuals, two from each family, presented with fatal neonatal lactic acidemia and died within a few days of birth. All were homozygous for this allele.

Two additional mutations in NDUFS6 were identified, first in 2017 [34] and then again in 2019 [35] when a more extensive analysis was done. Both were characterized as having Leigh syndrome, and the individuals were compound heterozygous, carrying both mutant alleles, c.309 + 5 G > A and c.343 C > A (p.Cys115Arg). The individual analyzed in 2019 lived for eleven months. It was determined that the splicing error led to a transcript missing exon 3, but that a small amount of normally spliced form was present. Complex I assembly was very limited, as indicated by BN gel electrophoresis. The mutation of Cys115 to Arg or Tyr could disrupt the folding of the protein and its binding to the matrix arm, which could have led to the loss of the FeS cluster N4, as was shown to happen in the fungal *Y. lipolytica* enzyme [29].

#### 2.2.3. NDUFA9

The gene for NDUFA9 is located on chromosome 12. It is encoded as a protein of 377 amino acids, about 42.5 kDa, but the N-terminal 35 amino acids are cleaved upon entry to the mitochondrion. The protein contains two domains, one being a Rossmann fold with an NADP^+^ bound to the C-terminal ends of the parallel beta-strands of the central sheet. A second domain, which contacts the NADP^+^, is largely alpha-helical. The second domain is largely C-terminal, and the Rossmann fold is largely N-terminal, except for two helices have been domain-swapped (see Figure 5). This protein is related to a family of short chain dehydrogenases, as first identified by Fearnley and Walker [36]. It is also one of the proteins that undergoes conformational changes in the active/deactive transition, as seen in mouse (PDB id = 6g2j, 6g72) [37] and sheep open and closed structures [38]. NDUFA9 is found at the junction of the Q-module and the membrane arm. It primarily has contact with core subunits NDUFS3 and NDUFS7, as well as with NDUFS4, NDUFS6, and NDUFA6. It also has minor contact with the core subunits ND1, ND3, ND6, NDUFS1, and NDUFS8. There is no evidence that this subunit exhibits any enzyme activity.

A human cell line was established with a knockout of NDUFA9 using transcription activator-like effector nucleases (TALEN) technology [39]. A clear assembly defect was discovered in which an 880 kDa complex was transiently seen, but a more stable 600 kDa complex was later found. The latter complex had the membrane arm subunits and some of the Q-module subunits of the matrix arm. In a more comprehensive study of all supernumerary subunits [11], the same group later established that levels of N-module subunits decreased the most in cell lines with the NDUFA9 knockout. The offered interpretation was that NDUFA9 is important for stabilizing the binding of the N-module to the Q-module during assembly. The complex seen in cells with the NDUFA9 knockout was smaller than those seen when NDUFS4 or NDUFS6 were knocked out, indicating that the Q-module was also destabilized.

Two missense mutations have been discovered in patients. The first was p.Arg321Pro, a homozygous mutation found in a boy of consanguineous parents [40]. He had complications in respiration after birth, with lactic acidosis and vision and hearing loss. He died after one month of respiratory failure, and it was classified as Leigh syndrome. An analysis of cultured fibroblasts showed very low levels of NDUFA9, and several other subunits, as well as essentially no assembled complex I in BN gel electrophoresis. Arg321 is found in the NADP^+^ binding pocket near the nicotinamide end but not in contact with it. It does not contact any other subunits, as it is completely buried. Therefore, it is likely that the substitution to Pro would disrupt the folding of the subunit and render it unable to properly bind to the assembling complex I.

The second discovered mutation also involved a homozygous Arg mutation, p.Arg360Cys [41]. This patient first exhibited symptoms of dystonia at age seven, leading to a loss of speech and becoming wheelchair-bound. His conditions stabilized in adulthood. A biochemical analysis did not reveal any metabolic disease, but an MRI of his brain revealed atrophy consistent with Leigh syndrome. An analysis of fibroblast mitochondria revealed reduced levels of complexes I and IV by BN gel electrophoresis, as well as reduced levels of several complex I subunits by immunoblotting. Complex I activity was 17–61% of control samples. Complementation with wild-type NDUFA9 confirmed that this mutation was causative for the reduced complex I activity. Arg360 is near the C-terminus of the subunit and is sandwiched between Trp361 of NDUFA9 and Tyr78 of ND6, a core subunit. A similar arrangement is seen in the mouse structure of the active conformation [37], except that Glu80 of ND6 is also involved. This junction between NDUFA9 and ND6 appears to be disrupted in the deactive conformation, as shown in the mouse structure [37] in which the C-terminus of NDUFA9 is not visible and is perhaps disordered. A similar situation has been seen in the higher resolution structures from sheep [38] in which the loops between NDUFA9 and ND6 are formed in the closed structures (PDB id = 6zko and 6zkc), while these loops, including Arg360 are disordered in the open structures (PDB id = 6zkd, 6zke, 6zkf, 6zkp, and 6zkr). Therefore, it is possible that even if the p.Arg360Cys subunit can assemble, it might be less able to undergo conformational changes during turnover, and so activity would decrease.

### 2.3. ND1-Module Subunits

**Figure 6:**
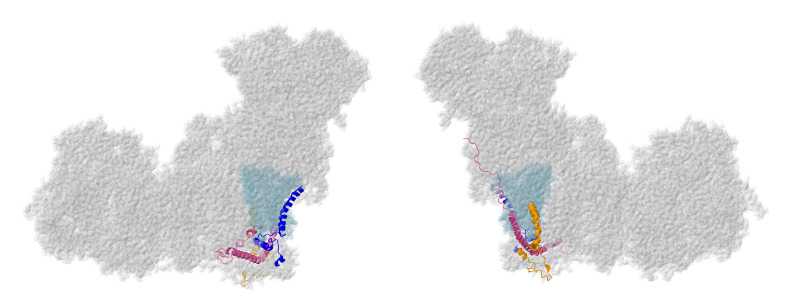
Location of NDUFA1, NDUFA3, and NDUFA13. Most of Complex I is colored gray. Core subunits in the ND1-modules are colored light blue (ND1). NDUFA1, NDUFA3, and NDUFA13 are shown in ribbons, with NDUFA1 colored blue, NDUFA3 colored orange, and NDUFA13 colored pink. The two views are rotated 180° relative to each other. The structure is from PDB id = 5xtd [9].

Three membrane subunits from the “heel” of complex I are described next: NDUFA1, NDUFA3, and NDUFA13, as shown in Figure 6. These subunits are part of the ND1-module for assembly. Each crosses the membrane once. NDUFA3 interacts with the transmembrane domain of NDUFA13, while NDUFA1 interacts with NDUFA13 in the IMS.

#### 2.3.1. NDUFA1

**Figure 7:**
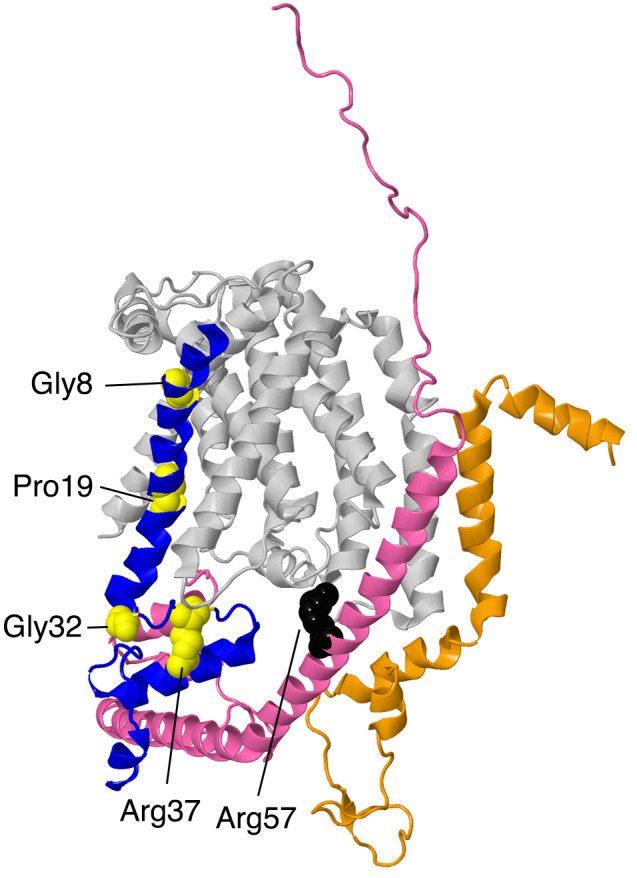
Structural features of ND1-module subunits. The proteins are portrayed in ribbons. ND1 is in the background and colored light gray. NDUFA1 is colored blue with the sites of 4 mutations, Gly8Arg, Pro19Ser, Gly32Arg, and Arg37Ser, shown in space-filling and colored yellow. NDUFA3 is shown in orange. No point mutations have been discovered yet. NDUFA13 is colored pink. The site of one mutation, Arg57His, is shown in space-filling and colored black.

The NDUFA1 gene is located on the X chromosome. The NDUFA1 subunit, also known as MWFE, contains 70 amino acids and has a mass of 8.0 kDa. It is a single-pass transmembrane protein that lies at the junction of the membrane and matrix arms of complex I. The N-terminus lies at the matrix surface. The protein consists of two separate domains: residues 1–31 form an alpha-helix that is situated in a groove between the first and seventh transmembrane helices of core subunit ND1. The C-terminal domain lies in the IMS and contains an alpha-helix (residues 42–56) and a short 3–10 helix (residues 65–70). This domain primarily contacts NDUFA8 (see Figure 7). A Ser residue is phosphorylated at position 55, but the significance of this modification remains unknown [42]. In a knockout strain of cultured human cells, the loss of NDUFA1 resulted in a reduced expression of complex I, and it migrated as a smaller-than-normal-sized complex in BN gel electrophoresis [11]. NDUFA1 mainly contacts core subunit ND1 and supernumerary subunit NDUFA8, but it also weakly contacts core subunit ND6 and supernumerary subunits NDUFS5, NDUFS8, and NDUFA13.

Four clinical mutations have been identified in this subunit: p.Gly8Arg, p.Arg37Ser, p.Gly32Arg, and p.Pro19Ser, and so would be hemizygous in males. The p.Gly8Arg mutation was identified in two half-brothers diagnosed with Leigh syndrome who both died in infancy [43]. The brothers had different biological fathers but shared a mother who carried the p.Gly8Arg mutation. The older brother developed psychomotor retardation at nine months and generalized hypotonia and choreoathetosis. At 19 months of age, brainstem lesions were observed, and the patient died from cardiorespiratory failure. The younger brother presented with axial hypotonia, vertical rolling nystagmus, choreoathetosis, and bilateral lesions. He developed respiratory insufficiency at 13 months of age and died at 14 months of age from cardiorespiratory arrest. The family pedigree shows that three maternal uncles had died of an unknown disease. The severity of the disease is reflected in the structural importance of the Gly8 residue on NDUFA1. Gly8 lies in the conserved hydrophobic N-terminal region of NDUFA1. Gly8 is in loose contact with residues Thr23, Lys26, and Leu43 in core subunit ND1. The substitution of the glycine for an arginine would be disruptive to interactions in this region.

The p.Arg37Ser mutation was identified in a boy who was diagnosed with generalized hypotonia, myoclonic epilepsy, and cerebellar atrophy [43]. His clinical evolution stabilized, and he was still living at 10 years old. His mother was a heterozygous carrier for the p.Arg37Ser mutation. BN gel electrophoresis demonstrated a low level of complex I formation, and muscle cells showed 15–30% of the normal level of complex I activity. The loss of a positive charge may disrupt the local structure or affect the phosphorylation of the subunit at Ser55. Arg37 contacts two residues from supernumerary subunit NDUFA8: it forms a hydrogen bond with Ser22 and is in contact with Gln92. It is also located near Asp90 and Gly93 from NDUFA8. Furthermore, according to mouse active (PDB id-6g2j) and deactive (PDB id = 6g72) structures [37], the residue may engage in different intermolecular interactions depending on the state of complex I, although this was not seen when comparing open and closed structures from the ovine enzyme [38].

The p.Gly32Arg mutation has been well-studied. Three separate clinical studies have been performed on this mutation. The first study [44] focused on two male patients who were maternal cousins from the same healthy non-consanguineous family. One patient experienced deterioration of motor and verbal skills at age four and an unsteady gait, retinitis pigmentosa, and cerebellar atrophy at age seven. His cousin developed an ataxia and proximal muscle weakness at age five and myoclonic seizures and bilateral sensorineural hearing loss at age 10. Both probands had mothers who were heterozygous carriers. Both men had lived into their thirties when the study was conducted. A six-year-old boy was also found to carry the p.Gly32Arg mutation [45]. Though the age of onset was not recorded, he experienced episodic neuroregression and encephalopathy but was seizure-free. His family pedigree was unknown. This finding is in concordance with the fact that the p.Gly32Arg mutation is not as deleterious as the p.Gly8Arg mutation. In 2011, a female patient with a heterozygous p.Gly32Arg mutation was identified [46]. She did not show signs of deficiency until 11 months of age, when she developed an X-linked respiratory chain deficiency in skeletal muscle tissue. She developed frequent upper airway infections and experienced somnolence and muscle hypotonia during these illnesses. However, at age five, she showed nearly normal psychomotor development. Though there was only a 25% expression of the normal allele in her skeletal muscle tissue, she showed a relatively mild clinical phenotype. It is hypothesized that X-inactivation due to selection advantage may have favored the expression of the normal allele. Neither her mother nor her father had the p.Gly32Arg mutation, suggesting that the mutation arose spontaneously. The fact that all four patients carrying the p.Gly32Arg mutation survived into adulthood may be attributed to the fact that Gly32 in NDUFA1 lies in the membrane near the cytoplasmic side and is not located at the interfaces of NDUFA1 and other subunits.

In 2014, a patient diagnosed with Leigh syndrome and mitochondrial respiratory chain disorder (MRCD) was discovered to have the p.Pro19Ser mutation [47]. The age of onset was five years old, but before 10 months of age, the boy had already experienced hypotonia, nystagmus, generalized epilepsy, and high blood lactate and pyruvate levels. Pro19 in NDUFA1 is a highly conserved in vertebrates and lies in the N-terminal membrane spanning helix (Pro7–Arg28). The residue contacts Leu9, Pro12, and Met91 of core subunit ND1 in the membrane region of the bilayer, and the substitution of the polar serine for proline could be deleterious.

#### 2.3.2. NDUFA3

The NDUFA3 gene is located on chromosome 19. The NDUFA3 protein is a 9.3 kDa protein that consists of 83 amino acids. It is a single-pass protein located in the inner mitochondrial membrane. It lies in the junction between the membrane and matrix arms of complex I and contains a kinked transmembrane alpha-helix domain that spans residues 17–35 and 37–49. During processing, the initiator methionine is cleaved, and Ala at position 2 is N-acetylated by analogy with the bovine enzyme [48]. NDUFA3 contacts core subunits ND1, ND3, and NDUFS8. Additionally, it contacts supernumerary subunits NDUFA8 and NDUFA13 (see Figure 7). In a knockout strain of cultured human cells, the loss of NDUFA3 resulted in a reduced expression of complex I and migrated as a smaller-than-normal-sized complex in BN gel electrophoresis [11]. An earlier study demonstrated the role of NDUFA3 in the assembly of the Q-module of complex I [49].

Though no clinical mutations in NDUFA3 have been identified yet, it has been shown that the full deletion of the NDUFA3 gene, along with other genes on chromosome 19, results in retinitis pigmentosa (RP), a disorder caused by photoreceptor cell degeneration. It is characterized by vision loss following night-blindness. In 2006, a family was identified as carrying a 30 kb deletion in chromosome 19 [50]. The family members afflicted with RP contained at least one copy of chromosome 19 that lacked a region containing genes NDUFA3, TFPT, PRP31, and the OSCAR promoter. The ages of onset of the RP symptoms varied from 3 to 30 years of age. Surprisingly, the patients were not afflicted by any disease other than RP. From this study, the absence of symptoms relating to complex I deficiencies suggests that only one copy of the gene for NDUFA3 may be sufficient for complex I activity.

Similarly, in 2011, another family was discovered to harbor a 112 kb deletion that encompassed the PRP31 gene and five of its upstream genes: TFPT, OSCAR, NDUFA3, TARM-1, and VSTM-1 [51]. Only one family member, a 33-year-old female, was diagnosed with RP. Once again, the RP patient showed no signs of complex I deficiency, and the authors suggested that one copy of NDUFA3 was sufficient for complex I activity.

#### 2.3.3. NDUFA13

The NDUFA13 gene is located on chromosome 19. NDUFA13 is a unique complex I subunit because it functions as a cell-death regulatory protein, GRIM-19, as well. It has been suggested that GRIM-19 regulates STAT3, a signal transducer and transcription activator, and is involved in interferon-β- and retinoic acid-induced cancer cell death [52]. For this reason, although NDUFA13 is mainly found in the inner mitochondrial membrane as a single-pass membrane protein, it can also be translocated to the nucleus to serve its apoptotic purpose. NDUFA13 is a 16 kDa protein synthesized as a 144-residue polypeptide, but the initiator methionine is removed during processing. It has a transmembrane alpha helix that spans residues 30–51 (see Figure 7). NDUFA13 has a longer alternate isoform that exists as a 222-residue polypeptide. NDUFA13 shares a large contact surface area with core subunits ND1, ND6, and NDUFS2, NDUFS5, NDUFA3, and NDUFA8. It weakly contacts ND3, NDUFS8, and supernumerary subunits NDUFA1 and NDUFA7. In a knockout line of cultured human cells, the loss of NDUFA13 resulted in a reduced expression of complex I, and it migrated as a smaller-than-normal-sized complex in BN gel electrophoresis [11]. Furthermore, when homozygous GRIM-19-deficient mice were generated, embryonic lethality resulted [53].

At least two diseases have been associated with mutations in NDUFA13. In 2015, a pair of sisters were discovered to harbor the first pathogenic germinal mutation identified in the short isoform one of NDUFA13, p.Arg57His (c.170 G > A in exon 2) [54]. Both sisters were homozygous for the mutation and presented with early onset hypotonia, dyskinesia, auditory neuropathy, and severe optic neuropathy. The patients were siblings who had been born from a consanguineous marriage. Their parents and elder sister were heterozygous for the p.Arg57His mutation and asymptomatic. The sisters were still living in 2015, the elder sister being 12 years old at the time. Though the sisters experienced early-onset neurological symptoms, the progression of neurological symptoms has been slow. Arg57 is a highly conserved residue across species. Upon biochemical analysis, NDUFA13 expression was reduced by a mean of 70% in the two sisters. Additionally, NDUFA9 and NDUFB8 expression was reduced by 95% and 90%, respectively. Due to the absence of intermediary complex I structures in the BN gel electrophoresis, the p.Arg57His mutation was concluded to cause major instability in NDUFA13 and prevent complex I assembly. It was suggested that the varied clinical symptoms in these sisters could be attributed to the dual function of NDUFA13/GRIM-19.

Somatic mutations in the long isoform two of NDUFA13 have been implicated in Hurthle cell thyroid tumors. Isoform two of NDUFA13 has 222 amino acids instead of 144 amino acids like isoform one. In 2005, four unrelated patients with Hurthle cell thyroid carcinomas were found to harbor four different heterozygous missense mutations that affected the N-terminal sequence of NDUFA13 [55]. Because the patients’ transcripts arose from an alternate reading frame and coded for isoform two, a structural analysis of these mutations cannot be performed.

### 2.4. LYR Family Subunits

**Figure 8:**
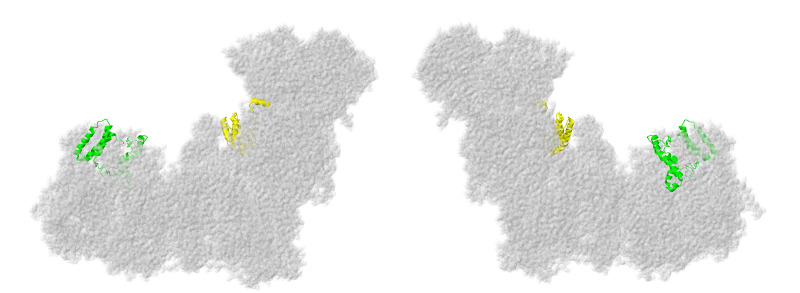
Location of NDUFA6 and NDUFB9. Most of Complex I is colored gray. They are shown in ribbons, with NDUFA6 colored yellow and NDUFB9 colored lime. The two views are rotated 180° relative to each other. The structure is from PDB id = 5xtd [9].

Two subunits from the LYR family are described next: NDUFA6 and NDUFB9, as shown in Figure 8. These proteins have the LYR sequence motif, which helps them bind to one of the two acyl carrier proteins, NDUFAB1. They also bind the substrate 4′-phosphopantethiene for the acyl carrier proteins. They are found on the matrix side of the membrane arm.

#### 2.4.1. NDUFA6

**Figure 9:**
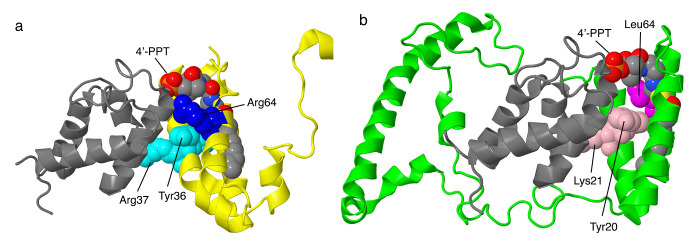
Structural features of the LYR proteins, NDUFA6 and NDUFB9. (**a**) NDUFA6 (colored yellow) is shown with its partner NDUFAB1 (colored gray), known as one of the two acyl carrier proteins (ACPs). NDUFA6 binds the 4′-phosphopantethiene (4′-PPT) analog substrate for the ACP. Tyr36 and Arg37 of the LYR motif are shown in space-filling and colored cyan. The site of the Arg64Pro mutation is shown in space-filling and colored blue. (**b**) NDUFB9 (colored lime) is shown with its partner NDUFAB1 (colored gray), the other one of the two ACPs. NDUFB9 binds the 4′-PPT analog substrate for the ACP. Tyr20 and Lys21 of the LYR motif are shown in space-filling and colored pink. The site of the Arg64Pro mutation is shown in space-filling and colored magenta.

The gene for NDUFA6 is located on chromosome 22. The encoded protein has 128 amino acids, with a mass of about 15.1 kDa. This subunit is found on the matrix arm. Its tertiary structure resembles a four-helix bundle in which the fourth helix is pulled away from the other three (see Figure 9a). Helices 1 and 2 interact with one of the two NDUFAB1 subunits, which are also known as the acyl carrier proteins and presumed to be involved in fatty acid biosynthesis. The short C-terminal helix interacts with core subunit NDUFS1, while core subunit NDUFS3 and supernumerary subunit NDUFA9 interact with both the N-terminal helices and the extended C-terminus. Thus, NDUFA6 bridges the N- and Q-modules. NDUFA6 is a member of the LYR family [56], whose members are known to interact with large mitochondrial complexes. Leu35 of the LYR motif fits inside the bundle of three helices, while Tyr36 and Arg37 contact NDUFAB1. In knockout cell lines, the assembly of full-size complex I was diminished, as seen in BN gel electrophoresis, and the levels of all subunits associated with the N-module of the peripheral arm were decreased by two-fold [11].

Mutations in NDUFA6 have been described for four individuals, though only recently [57]. The most severe of the illnesses occurred in an individual with compound heterozygous mutations: p.Arg64Pro and c.265 G > T, a nonsense mutation at Glu89. An analysis of tissue demonstrated almost no fully assembled complex I and almost no in-gel activity in BN gel electrophoresis. This individual died after about two days. Tissue from two other individuals was analyzed and showed low levels of assembled complex I with activity in BN gel electrophoresis. Residue Arg64 is ion-paired with Asp111 of NDUFAB1 and is near Asp92 of NDUFAB1 and Tyr36 of the LYR motif of NDUFA6. The adjacent Ser112 of NDUFAB1 is part of the catalytic site of the acyl carrier enzyme and is bound by a substrate in the human structure (PDB id = 5xtd) [9].

The second individual was homozygous for a two-nucleotide deletion at Glu111, c.331_332del, resulting in 35 altered amino acids before a stop codon appeared [57]. This transcript was found at normal levels, thus indicating the lack of nonsense-mediated decay, presumably because it occurs in the last of three exons near the normal termination codon. If this transcript was translated, the protein would lack the C-terminal alpha-helix that interacts with core subunit NDUFS1. The third individual was homozygous for a c.3 G > A, substitution in the start codon. It was suggested that a transcript starting from an upstream Met codon, as predicted by another isoform, might have permitted some expression of a functional protein. Both individuals showed low levels of fully assembled complex I with activity, and their outcomes were only somewhat better than the first, with abnormal white matter in the brain, a loss of movement and vision, and seizures. The fourth individual was compound heterozygous with two frameshift mutations: p.Met104Cysfs*35 and p.Leu119Tyrfs*20. This child died in infancy, and no biochemical analyses were performed.

#### 2.4.2. NDUFB9

The gene for NDUFB9 is located on chromosome 8. It is encoded as a protein of 179 amino acids, with a mass of about 21.8 kDa. The N-terminal Met is likely removed, and other possible modifications include the acetylation of Ala2 [58] and the phosphorylation of Ser85 [59]. NDUFB9 is a peripheral protein that is bound to the matrix side of core subunit ND5, with significant contacts to NDUFAB1, one of the acyl carrier proteins (see Figure 9b), and to NDUFB3, NDUFB4, NDUFB5, and NDUFB6. The protein is U-shaped, with each arm consisting of a three-helix bundle and pointing away from ND5. NDUFB9, along with NDUFA6, is a member of the LYR protein family, characterized by a Leu–Tyr–Arg motif near the N-terminus. In the case of NDUFB9, the motif is Leu19–Tyr20–Lys21, and it is found in the N-terminal alpha-helix. The acyl carrier protein NDUFAB1 fits between the two helical domains and binds to Tyr20 and Lys21 of this motif. In a cell line in which NDUFB9 was knocked out, complex I failed to assemble [11].

In 2012, a large-scale mutation-screening identified two patients who carried NDUFB9 mutations [60]. One patient, who presented with lactic acidemia and muscular hypotonia, was homozygous for the missense p.Leu64Pro. This residue is found in the third helix and packs against the first helix in the N-terminal three-helix bundle. Complex I activity was about 39% of the normal activity. After lentiviral rescue with a wild-type NDUFB9 gene, both complex I activity and levels of other complex I subunits were restored to normal levels. A second individual was found to carry the heterozygous mutation Arg47Leu, but this mutation was not found in a sibling with similar symptoms and could not be rescued by the complementation with a wild-type NDUFB9 gene. Therefore, it was concluded that illness was due to another gene.

### 2.5. Subunits of the Twin C–X_9_–C Family in the IMS

**Figure 10:**
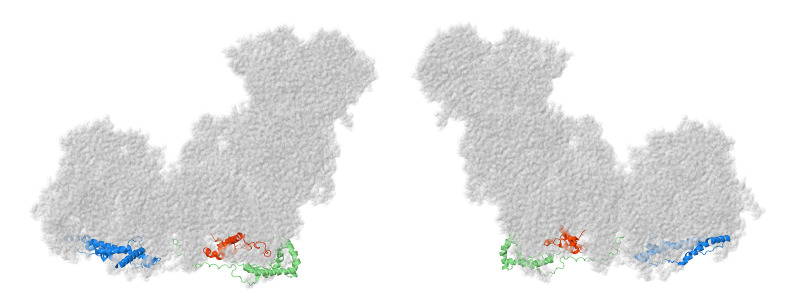
Location of NDUFS5, NDUFA8, and NDUFB10. Most of Complex I is colored gray. NDUFS5, NDUFA8, and NDUFB10 are shown as ribbons, with NDUFS5 colored red, NDUFA8 colored green, and NDUFB10 colored blue. They are found facing the intermembrane space. The two views are rotated 180° relative to each other. The structure is from PDB id = 5xtd [9].

Complex I has four proteins that are members of the twin C–X_9_–C family and three with identified mutations that are described next: NDUFS5, NDUFA8, and NDUFB10, as shown in Figure 10. All are found in the IMS. They are substrates of the IMS oxidoreductase CHCHD4, also known as hMia40, which is essential for the export of FeS clusters from the mitochondrial matrix [61].

#### 2.5.1. NDUFS5

The gene for NDUFS5 is found on chromosome 1. The protein contains 106 amino acids, with a mass of 12.5 kDa. In cultured human cell lines, the knockout of NDUFS5 completely eliminates the assembly of complex I [11]. Though this protein was initially identified with the iron–sulfur protein fraction of bovine complex I, it is now known to be localized to the IMS and does not contact any proteins with FeS clusters. NDUFS5 is bound to the surface of the membrane and lacks transmembrane helices. It has a limited tertiary structure and includes a helix–coil–helix with two C–X_9_–C sequence motifs that form two disulfides between the two alpha-helices at residue pairs 33–66 and 43–56. Another alpha-helix is formed by residues 70–86, but the rest of the protein has little regular secondary structure. The N-terminal domain (2–30) primarily contacts core subunits ND2 and ND4L, and supernumerary subunit NDUFB5, while the C-terminal domain (70–106) primarily contacts core subunit ND6, and NDUFB13.

One missense mutation in NDUFS5 has been reported in a compound heterozygous individual, p.Pro96Ser [22]. This individual has a second mutation in core subunit NDUFS8, p.Arg2Cys, of unknown consequence. These mutations were identified in a cohort of 103 patients with complex I deficiency. Pro96 in NDUFS5 is highly conserved among vertebrates and appears as the third Pro in an unusual sequence motif of Pro-Pro-Pro-His-His near the C-terminus. In human complex I (Video S1), Pro96 contacts Thr129 of NDUFA13. There is a nearby ion pair between Lys101 of NDUFS5 and Glu89 of NDUFA13. Somewhat different interactions can be seen in the mouse complex I in both active and deactive conformations [37]. Therefore, it seems likely that this missense mutation would disrupt local structure, but it is not clear that it would be deleterious to complex I assembly. The mutation in NDUFS8, p. Arg2Cys, should also be considered, as it might be defective, for example, in translation initiation.

#### 2.5.2. NDUFA8

**Figure 11:**
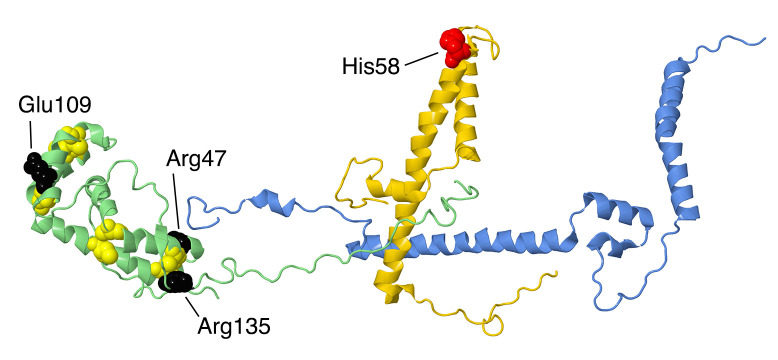
Structural features of NDUFA8, a member of the twin C–X_9_–C. The proteins are portrayed in ribbons. NDUFA8 is colored green, NDUFC2 is colored gold, and NDUFB5 is colored blue. In NDUFA8, the eight Cys residues that form disulfide bonds are shown in space-filling and colored yellow. The sites of three mutations, Arg47Cys, Glu109Lys, and Arg135Gln, are shown in space-filling and colored black. NDUFB5 makes contact near Arg47, and both NDUFB5 and NDUFA8 contact NDUFC2. The site of one mutation in NDUFC2, His58Leu, is shown in space-filling and colored red.

The gene for NDUFA8 is located on chromosome 9. The protein has 172 amino acids with a mass of about 20 kDa, and the initial methionine is cleaved. NDUFA8 is found in the IMS and has large contact surfaces with supernumerary subunits NDUFA13, NDUFA3, and NDUFA1, and it is near core subunit ND1. It has two pairs of alpha helices, each having two pairs of Cys residues that likely form disulfides (see Figure 11). They have characteristic spacings of either 9 or 11 amino acids between the cysteine residues. In the human structure (PDB id = 5xtd), not all disulfides are shown as formed, but it is likely that they do form in the IMS: C36–C66, C46–C56, C78–C110, and C88–C100. In the mouse structures (PDB id = 6g2j and 6g72), they are shown as disulfides [37]. The N-terminal residues 2–22 are extended and interact with NDUFA13, NDUFS5, and NDUFA1. The C-terminal region extends from residues 113 to 172 without a regular secondary structure. It makes an interesting junction with NDUFC2 and NDUFB5 in a conserved multi-centered ion pair including Arg166 of NDUFA8, Asp86 of NDUFC2, and Glu153 of NDUFB5.

At least three missense mutations have been described in NDUFA8. The p.Arg47Cys mutation was described in a patient that was homozygous, having received a mutant allele from each parent, who were heterozygous and asymptomatic [62]. This individual presented with developmental delay and epilepsy, and they were bed-ridden by age 26. Fibroblasts showed reduced levels of NDUFA8, as well as other complex I subunits, and reduced complex I activity. The mutation creates an additional Cys residue next to Cys46, which possibly leads to incorrect disulfide bond formation. In addition, Arg47 has multiple interactions within the NDUFA8 subunit, as well as with NDUFA13 and NDUFB5, which would be disrupted in the mutant p.Arg47Cys.

The second individual was compound heterozygous with p.Glu109Lys in NDUFA8 and p.Ala224Val in core subunit NDUFS2 [63]. The transcript for the mutant NDUFA8 subunit was not found, and so the mutation might have affected its stability. Ala224 in the core subunit NDUFS2 is highly conserved and is found near Cys 153 and 160 of a FeS cluster, and so this substitution might also have been deleterious. The infant showed neonatal hypotonia and died at two months.

The third individual was found in a screen of patients with reduced complex I levels [22]. Two potential deleterious mutations were discovered. First was a heterozygous mutation p.Arg135Gln in NDUFA8, and second was a homozygous mutation p.Leu229Pro in C20orf, a spindle assembly factor for microtubles. Since the individual showed decreased levels of complex I activity, the NDUFA8 mutation was implicated, but it was not clear whether the spindle assembly factor had any impact on complex I levels. Arg135 is highly conserved, and it makes an ion pair with Glu59, also a conserved amino acid of NDUFA8. Glu59 is near the disulfide formed by Cys46 and Cys56. This ion pair is exposed on the IMS and does not contact any other subunits.

#### 2.5.3. NDUFB10

The gene for NDUFB10 is located on chromosome 16. The NDUFB10 protein has 172 amino acids and a mass of 20.8 kDa. It lacks a mitochondrial import sequence. It is one of four IMS proteins found in complex I, and all have four cysteine residues that form two disulfide bonds. In NDUFB10, two cysteine residues, Cys78 and Cys107, are found near the ends of a long alpha-helix (M77–E111). These cysteine residues form disulfides with nearby cysteines, connected to the long helix by loops of 6–11 residues. Cys78 forms its disulfide with Cys71, and Cys107 forms its disulfide with Cys119. In addition, there is another cysteine, Cys145, conserved among vertebrates, and of unknown significance. The import of NDUFB10 is dependent upon its oxidation by CHCHD4/Mia40 and its formation of correct disulfide bonds [64]. NDUFB10 also contains three additional alpha-helices, two C-terminal ones and one N-terminal one. These extensions allow it to contact many subunits in the vicinity of ND4 and ND5. It primarily contacts NDUFB5, NDUFB6, and NDUFB11, with lesser contact to ND4, ND5, and NDUFC2. In an analysis of cultured cells, the knockout of NDUFB10 resulted in a loss of complex I assembly and, especially, the ND4/5 modules.

One individual with mutations in NDUFB10 was studied [64]. She was compound heterozygous with a paternally inherited termination codon at Glu70 of NDUFB10 and a maternally inherited p.Cys107Ser substitution. This infant presented with lactic acidosis and cardiomyopathy and survived for only about one day. An analysis revealed reduced levels of complex I activity, reduced levels of in-gel assays of complex I in BN gels, and the presence of the Cys107Ser protein in the cytosol. The transcript of the nonsense allele was not found by RT-PCR. Cys107 is not near any other subunits, the closest being NDUFB5, NDUFB6, and NDUFB11. Given the healthy status of the parents, it appears that the Cys107S mutant does not have a dominant negative phenotype. Rather, it primarily does not enter the mitochondrion, and if another allele is available, complex I can assemble without difficulty.

### 2.6. Subunits of the ND2-Module

**Figure 12:**
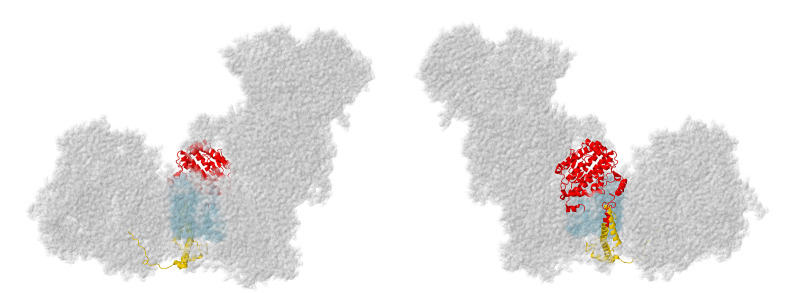
Location of NDUFA10 and NDUFC2. Most of Complex I is colored gray. Core subunits in the ND2-module are colored light blue (ND2). NDUFA10 and NDUFC2 are shown in ribbons, with NDUFA10 colored red and NDUFC2 colored gold. The two views are rotated 180° relative to each other. The structure is from PDB id = 5xtd [9].

The next two subunits to be described are members of the ND2-module: NDUFA10 and NDUFC2, as shown in Figure 12. NDUFA10 is a globular protein bound on the matrix side, while NDUFC2 is a membrane protein. These two subunits have contacts on the matrix side of the membrane.

#### 2.6.1. NDUFA10

**Figure 13:**
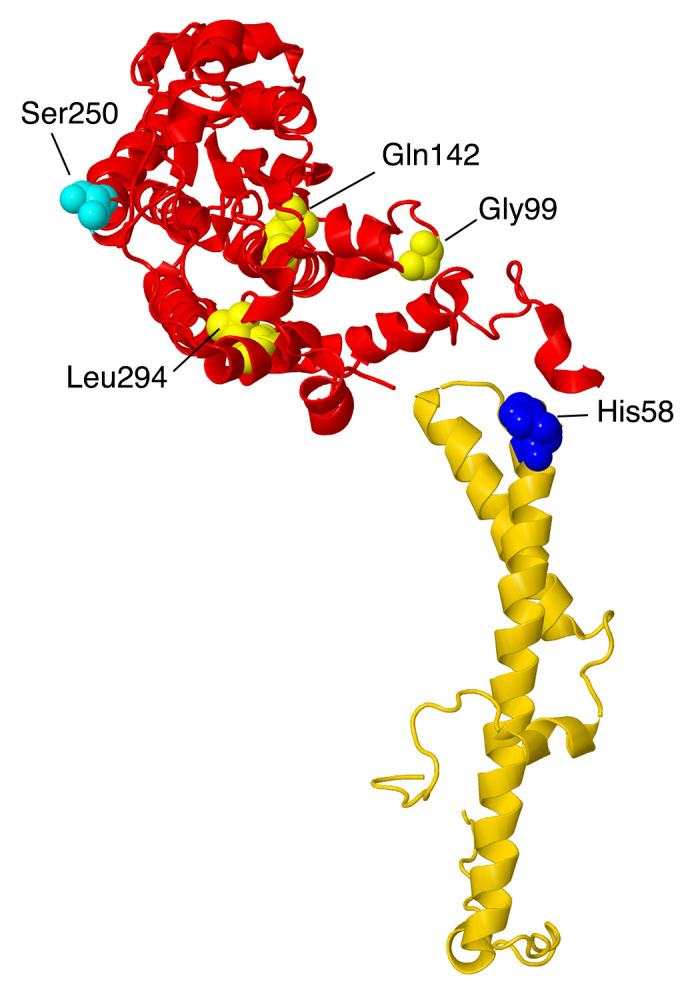
Structural features of two subunits from the ND2-module. NDUFA10, colored red, and NDUFC2, colored gold, are portrayed in ribbons. The site of one mutation, His48Leu in NDUFC2, is shown in space-filling and colored blue. The sites of three mutations in NDUFA10—Gly99Glu, Gln142Arg, and Leu294Pro—are shown in space-filling and colored yellow. The site of phosphorylation by PINK1, Ser250, is shown in space-filling and colored cyan. NDUFA10 likely binds an adenosine nucleoside, not shown.

The gene for NDUFA10 is found on chromosome 2. The encoded protein has 355 amino acids and a mass of about 40.8 kDa, with 35 N-terminal amino acids cleaved upon entry to the mitochondrion. Structurally, NDUFA10 has been identified as a member of the deoxynucleoside kinase (dNK) family [8], with the dNK domain (also known as the PF01712 family in the Pfam database), although it is unlikely to be an active enzyme. The protein is compact with a four-stranded parallel beta-sheet surrounded by numerous alpha-helices (see Figure 13). The human structure (PDB id = 5xtd) [9] did not reveal any ligands, but adenosine nucleotides have been found at the C-terminal ends of the beta-sheet in a pocket surrounded by alpha-helices in the mouse (PDB id-6g2j) [37] and sheep [65] structures. NDUFA10 is found on the matrix side of the membrane and primarily interacts with core subunits ND2 and NDUFS2. These interactions with the extended N-terminus of NDUFS2, and the matrix-side peripheral helices of ND2 are all centered over the C-terminal sector of ND2, which contains the broken helix that is part of the proton translocation pathway. NDUFA10 also makes limited contact with the N-terminal regions of NDUFC1, NDUFC2, and NDUFB11 on the matrix side.

In knockout human cell line BN gel electrophoresis, only faint bands of complexes containing primarily membrane subunits are seen [11]. Perhaps because of its contacts with a Q-module, core subunit NDUFS2, and a core membrane subunit ND2, along with subunits at the interfaces of ND2/ND4 (NDUFC1 and NDUFC2) and of ND4/ND5 (NDUFB11), it appears to be a key subunit in assembly. Residue Ser250, which is found on the matrix surface, was reported to be phosphorylated by the kinase PINK1 [66], a kinase that is known to be imported into the mitochondrial matrix space [66,67]. This is a conserved amino acid, and phosphorylation appears to be important for complex I activity.

Three reports of clinical mutations in NDUFA10 have been described. The first patient had compound heterozygous mutations including one allele in which the start codon was changed to GTG, and a second in which p.Gln142Arg occurred [68]. This individual showed developmental problems at 10 months and was eventually diagnosed with Leigh syndrome. It is likely that the first mutation would significantly impair the translation unless an alternative start codon were available. The second mutation is found in the interior of the protein, and so the Arg likely disrupts the packing of the protein, especially because of the positive charge.

The second patient was a boy with Leigh syndrome who, in a screen, was determined to have compound heterozygous mutations: p.Leu294Pro and c.383_384insTAA (p.Ser218delinslS) [27]. These were found to be inherited from the father and mother, respectively. The latter mutation would likely lead to a degraded mRNA or protein. The former mutation p.Leu294Pro resides on an alpha-helix at an interior location. The substitution of Leu by Pro might be disruptive, as this alpha-helix contacts two distinct regions of ND2. The immunoblotting of fibroblasts showed reduced levels of NDUFA10.

The third patient was initially diagnosed as having a nonlethal infantile mitochondrial disorder, and the mutation was discovered in a screen [69]. This individual developed brain lesions and was considered to have Leigh syndrome. His parents were third cousins, and DNA sequencing revealed him to be homozygous for p.Gly99Glu, while both parents were heterozygous for the same mutation. A deltoid muscle biopsy showed significant reduction of complex I activity (about 40–70% of normal levels). This amino acid is packed against the N-terminal amino acids of NDUFC1, and so the introduction of the negatively-charged Glu could disrupt the assembly of the membrane arm.

#### 2.6.2. NDUFC2

The gene for NDUFC2 is located on chromosome 11. The NDUFC2 protein is 119 amino acids, with a mass of 14.2 kDa. It is a double-pass transmembrane protein that contacts core subunit ND2, with both termini on the IMS side of the membrane. From the N-terminus, the protein enters the membrane in a non-helical stretch from Ser 19 to Arg29, and then residues 29–47 are alpha-helical. The second crossing of the membrane is alpha-helical from residues 56–97, extending into the IMS. NDUFC2 also contacts NDUFC1, NDUFB5, and NDUFA8 (see Figure 11), and it is part of the ND2 assembly module. NDUFC1 has a special role in sealing NDUFC2 from the lipid bilayer, with its single transmembrane helix parallel to the long helix of NDUFC2 and very limited contact to any other subunits in the membrane. The extension of the long alpha-helix into the IMS, residues 84–97, is the region that contacts NDUFA8 and NDUFB5 (see Figure 11). On the matrix side it contacts NDUFA10 (see Figure 13).

Mutations of NDUFC2 in three patients from two families were reported in 2020 [70]. The patients presented with symptoms of Leigh syndrome and reduced complex I activity but had different outcomes. In both families, the parents were healthy consanguineous first cousins and were heterozygous for the mutations. In one family, the mutation was a deletion of 22 base pairs near the C-terminus at residue His116, p.His116_Arg119delins21. This would cause a frame shift and elimination of the normal stop codon at position 120. It was not clear how long the new reading frame would be. The transcript of this gene was identified at low levels, but the immunoblotting of fibroblast samples was negative, suggesting the degradation of the protein or a lack of expression. Little or no complex I was seen in native gels. Complexome profiling found evidence of Q-module assembly and ND4-module assembly but little else of complex I subunits. The daughter had no seizures and survived until at least age six, while her brother passed away at three years of age.

In the second family, the mutation was p.His58Leu. This patient had normal transcript levels but only slightly more evidence of complex I assembly. His58 is found on the matrix side of the membrane and starts the long helix that crosses the membrane. It sits between Val44 of NDUFC1 and Trp353 of NDUFA10 and is very near the phosphate group of a bound lipid. In the mouse structure (PDB id = 6g2j), the corresponding His59 is even more tightly packed between NDUFC1 and NDUFA10 residues, suggesting that a Leu substitution could be deleterious. This child had impaired growth and seizures and passed away at eight months.

NDUFC2 has a unique position in complex I. It is embedded in the membrane near the ND2-ND4 junction but contacts two subunits that are embedded at distant sites, with long extension to NDUFC2: NDUFA8 is found at the “heel” of the complex I boot, in the IMS, while NDUFB5 is found at the junction of ND4-ND5. Interactions in this network of subunits appear to be essential for complex I assembly.

### 2.7. Subunits Form the ND4-Module

**Figure 14:**
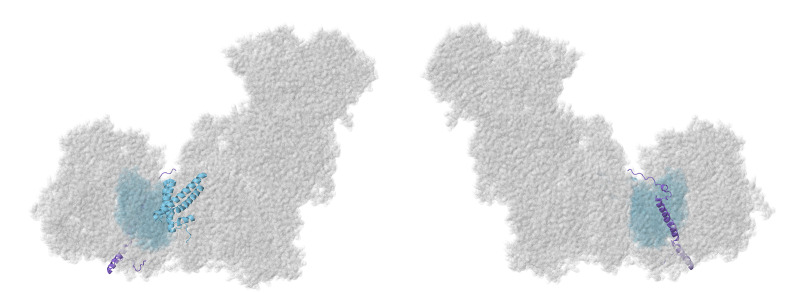
Location of NDUFA11 and NDUFB11. Most of Complex I is colored gray. Core subunits in the ND4-module are colored light blue (ND4). NDUFA11 and NDUFB11are shown in ribbons, NDUFA11 colored blue and NDUFB11 colored purple. The two views are rotated 180° relative to each other. The structure is from PDB id = 5xtd [9].

Two subunits from the ND4-module are described next: NDUFA11 and NDUFB11, as shown in Figure 14. Both are membrane proteins but are found on alternate sides of ND4 and do not contact each other.

#### 2.7.1. NDUFA11

The NDUFA11 gene is located on chromosome 19. NDUFA11 is a 14.7 kDa protein with four transmembrane helices and little exposure outside the membrane. NDUFA11 has two isoforms. The first isoform contains 140 amino acids after the cleavage of the initiator methionine and is N-acetylated at Ser2. It consists of five alpha helices: a short helix is found on the IMS side, followed by membrane spanning helices 3–11, 17–45, 48–82, 87–106, and 107–137. NDUFA11 contacts the core subunits ND2, ND4, and ND5, as well as the supernumerary subunit NDUFB5. The second isoform is rare and contains 228 amino acids. In a previous study, it was found that the knockdown of NDUFA11 by RNA interference (siRNA) in human cell culture led to partially assembled subcomplexes visualized by blue native gels [71]. It was concluded that NDUFA11 acts like an assembly factor in complex I. Furthermore, it was shown that, in a knockout strain of cultured human cells, the loss of NDUFA11 resulted in no expression of complex I in BN gel electrophoresis [11].

The first identified NDUFA11 clinical mutation, a G to A mutation at the exon-intron junction (exon 1-IVS1) donor splice site, c.99 + 5 G > A, was identified in 2008 [72]. Six patients from three unrelated families were products of consanguineous marriages and were found to be homozygous for the mutation. They were clinically affected with either fatal infantile lactic acidemia or encephalocardiomyopathy. Though heterozygotes were identified in two families, all family members besides the patients were healthy. The patient from Family A and the two patients from Family B had similar clinical presentations. They all developed severe metabolic acidosis and hyperlactatemia within 10–24 h of age. All three patients from Families A and B died from acidosis within 6–40 days of age. In Family C, three patients were homozygous for this splice mutation. They experienced slow psychomotor development, hypertrophy of myocardial walls, acidosis, and generalized brain atrophy. Two out of the three patients from Family C died at 18 months and four years of age, but the third patient from this family lived until at least six months of age. The presence of both wild-type and mutant mRNA transcripts in the patients’ fibroblasts indicates that the varied clinical presentation may have been caused by variable splicing that produced variable mutant/wild-type transcript ratios. They suggested that normal splicing would have produced a protein that was responsible for the detected activity, while alternative splicing would have yielded a nonfunctional protein.

In 2019, a two more NDUFA11 clinical mutations, p.Ala132Pro and p.Thr106Ile, were found in isoform two of NDUFA11 [73]. These mutations were found simultaneously in a compound heterozygote patient with mitochondrial myopathy. The patient developed late-onset symptoms of a neuromuscular disorder, bilateral hearing loss, saccadic eye movements, and proximal leg weakness. The good health of the patient’s offspring and the absence of neuromuscular disease in the patient’s family history suggested that these two mutations were spontaneous. In contrast to isoform one, NDUFA11 isoform two is rare and appears in skeletal muscle. Thus, a mutation in isoform two may only lead to mild muscular impairment. The structure of this rare isoform of NDUFA11 has not yet been determined, so we cannot confirm that the mutation causes a structural disruption within this subunit.

#### 2.7.2. NDUFB11

**Figure 15:**
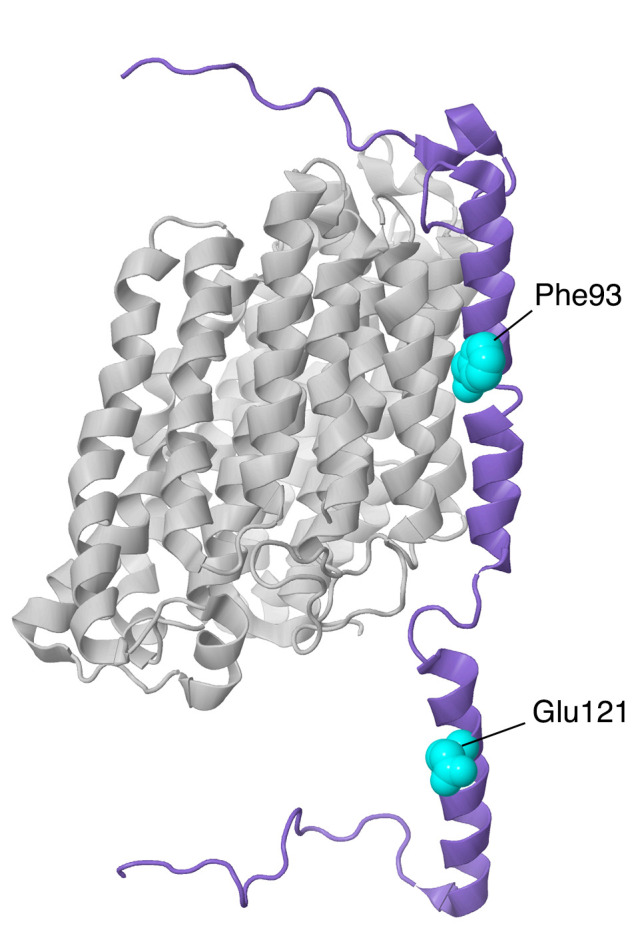
Structural features of NDUFB11 of the ND4-module. The proteins are shown as ribbons, with NDUFB11 colored purple and ND4 colored light gray. The sites of two mutations are shown in space-filling, Phe93 (a deletion) and Glu121Lys (in the IMS).

Similar to NDUFA1, the NDUFB11 gene is located on the X chromosome. It has three exons. It is synthesized as a 153 amino acid protein with a mass of about 17.3 kDa, but its first 29 amino acids serve as a transit peptide that is cleaved off during processing. NDUFB11 is a single-pass membrane protein with the N-terminus on the matrix side and one helix that spans residues 80–107, followed by another helix into the IMS of residues 115–132 (see Figure 15). Its N- and C-termini are extended and point in the same direction, like a letter C. It has a large contact surface with core subunit ND4 and NDUFB10, but it also weakly contacts core subunit NDUFS2, NDUFA10, NDUFB5, and NDUFC2. In a knockout line of cultured human cells, the loss of NDUFB11 resulted in almost no assembly of complex I [11]. Furthermore, NDUFB11 short-hairpin RNA (shRNA) knockdown in HeLa cells on complex I resulted in decreased expression of subunit NDUFB8, the failure of the membrane arm and the holocomplex to assemble, and decreased oxygen consumption. Moreover, NDUFB11 reduction was associated with decreased cell growth and increased apoptosis. For this reason, cell death caused by NDUFB11 mutations may be responsible for embryonic lethality in males and developmental defects in female patients [74].

Two studies conducted in 2015 found that nonsense mutations in NDUFB11 are associated with histiocytoid cardiomyopathy (histiocytoid CM) and microphthalmia with linear skin defects syndrome. Clinical characteristics of histiocytoid CM, an arrhythmogenic disorder, are incessant ventricular tachycardia, cardiomegaly, and sudden death within the first two years of life. The study did not expand on specific patient symptoms or survival. In the first study, two unrelated female patients with histiocytoid CM were identified to harbor de novo nonsense mutations in NDUFA11 [74]. The first patient had a mutation that changed Tyr108 to a stop codon (c. 324 T > G). The second patient had a mutation that changed Trp85 to a stop codon (c. 255 G > A). Both mutated residues are located in exon 2 of NDUFB11. The authors concluded that these de novo mutations result in a dominant haploinsufficient phenotype, which contrasts with the Mendelian recessive inheritance pattern of many complex I deficiencies.

In the second study, two female patients were discovered with microphthalmia and linear skin defects syndrome (MLS) [75]. MLS is an X-linked disease found exclusively in females and is embryonically lethal in males. Both patients presented with linear skin defects but not microphthalmia. Patient 1 was heterozygous for a de novo nonsense mutation, c.262 C > T in exon 2, that changed Arg88 to a premature stop codon. Besides MLS and histiocytoid CM, she experienced additional symptoms such as axial hypotonia, failure to thrive, oncocytic metaplasia, and histiocytoid cardiomyopathy. She died at six months of age from cardiac arrest. This mutation was also identified in a later study [76]. Patient 2 was heterozygous for an inherited one base-pair deletion, c.402delG in exon 3, that caused a p.Arg134Ser mutation and changed Val136 to a premature stop codon. She was observed to have corpus callosum agenesis and dilated lateral ventricles during the fetal stage and experienced seizures, cardiomyopathy, myopia, nystagmus, severe psychomotor developmental delay, and muscular hypotonia after birth. She was still living at seven years of age. Her mother was a healthy carrier of the mutation. Though the authors of the previous study [74] concluded that one copy of wild-type NDUFB11 was not sufficient for normal cell function, the existence of a heterozygous, healthy carrier of a NDUFB11 deletion contradicts this conclusion. We conclude that the variable outcomes for females might be due to mosaicism; phenotypic differences may depend on which tissues receive the wild-type NDUFB11 due to X-inactivation. After Patient 2′s birth, her mother was pregnant with another fetus who had the same frameshift mutation. The fetus had to be aborted due to severe intrauterine growth retardation. Only wild-type NDUFB11 transcripts were found in the fibroblasts and leukocytes of Patient 1 and Patient 2, indicating that the two mutations resulted in no transcript or expression of NDUFB11.

Another NDUFB11 mutation was found in 2016 [27]. A male patient who harbored a hemizygous de novo mutation (c.361 G > A, p.Glu121Lys) presented with lethal infantile mitochondrial disorder (LIMD), heart and respiratory failure, and complex I deficiency. There was no NDUFB11 expression in the patient’s fibroblasts, and he died 55 h after birth. Glu121 is a highly conserved residue. It lies on the intermembrane space side of complex I and sits between Arg124 and Tyr117 of the same subunit and near His50 of NDUFB10.

Additionally in 2016 [77], another mutation in NDUFB11 was discovered in five males from four families in a screen of patients with congenital sideroblastic anemia. The mutation was a three-nucleotide deletion, c.276_278del, p.F93del, occurring in three consecutive phenylalanine codons (TTC). These three Phe occur in the membrane helix between ND4 and NDUFB5 (see Figure 15). It is possible that the deletion could be partially accommodated by the chain of three consecutive Phe residues. The subjects ranged from 2 to 76 years of age, and many had associated symptoms of short stature, congenital optic atrophy, myopathy, and lactic acidosis.

### 2.8. Subunits from the ND5-Module

**Figure 16:**
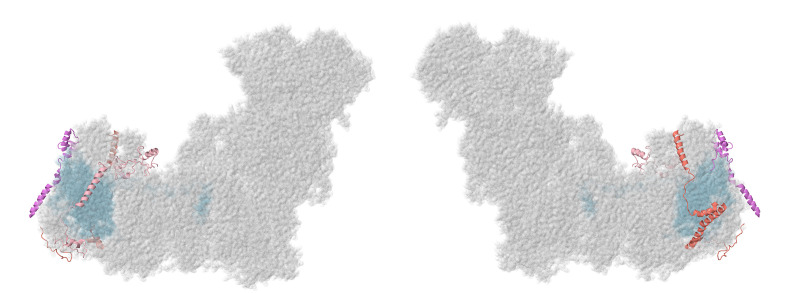
Location of NDUFB3, NDUFB6, and NDUFB8. Most of Complex I is colored gray. Core subunits in the ND5-module are colored light blue (ND5). NDUFB3, NDUFB6, and NDUFB8 are shown in ribbons, with NDUFB3 colored violet, NDUFB6 colored salmon, and NDUFB8 colored pink. The two views are rotated 180° relative to each other. The structure is from PDB id = 5xtd [9].

Finally, three subunits are next described from the ND5-module: NDUFB3, NDUFB6, and NDUFB8, as shown in Figure 16. These subunits do not interact directly with each other, but they serve to enclose the distal end of complex I on all sides. In particular, NDUFB8 clamps the lateral helix of core subunit ND5.

#### 2.8.1. NDUFB3

The gene for NDUFB3 is found on chromosome 2. The encoded protein has 99 amino acids with a mass of about 11.4 kDa. The N-terminal Met appears to be cleaved. NDUFB3 is found at the distal end of complex I near core subunit ND5. It has a small N-terminal domain composed of three alpha-helices on the matrix side, and the C-terminus forms an alpha-helix, residues 62–89, that crosses the membrane. NDUFB3 contacts NDUFB9 and NDUFAB1 on the matrix side, as well as NDUFB2 and core subunit ND5, primarily in the membrane region. In knockout cell lines, the absence of NDUFB3 was found to result in the near total loss of complex I assembly and a reduction in the level of subunits from both N- and ND5 assembly modules [11].

Two mutations have been identified in the gene for NDUFB3 among over 10 individuals. In a report from [78], p.Trp22Arg appeared as a homozygous mutation in a girl who exhibited intrauterine growth retardation and premature birth at 31 weeks. She died at four months of age. Very low levels of complex I activity (<15%) were measured in fibroblasts, and this phenotype was rescued by lentivector complementation. A second individual was identified with compound heterozygous mutations: p.Trp22Arg and p.Gly70X [79]. The level of complex I activity was <25% of normal, and the individual exhibited muscular hypotonia, developmental delay, and lactic acidosis. In fibroblasts, complex I activity could be recovered by the ectopic expression of wild-type NDUFB3, though not either mutant form. The health status of this individual was not reported. In contrast, two more recent reports have identified the p.Trp22Arg mutation in older and seemingly healthier individuals. Ten children, up to 10 years of age, were identified as homozygous carriers of p.Trp22Arg in NDUFB3 [80]. These individuals with short statures and prominent foreheads ranged from 10 months to 10 years in age, and their complex I activity levels ranged from 25 to 35% of normal levels. Various levels of assembled complex I were seen from these individuals using blue native gel electrophoresis. More recently, a 32-year-old previously diagnosed with non-alcoholic steatohepatitis was discovered to be homozygous p.Trp22Arg [81]. This individual had reduced levels of complex I activity (<30% of normal) and suffered from oculomotor dysfunction with optic nerve anomalies, episodes of lactic acidosis during surgical interventions, and progressive fatigue.

The latter two studies emphasized that the context of these two mutations in NDUFB3 appears to be significant. The level of complex I activity, and its assembly, is likely on the borderline between tolerable and deleterious. Trp22 is found in a conserved region of NDUFB3 on the matrix side of the membrane. The NH side chain of Trp is in position to H-bond to the OH side chain of Tyr85 of NDUFAB1. It also makes nonbonding interactions with Arg45 and Gln46 of NDUFB2. Therefore, the mutation to the positively charged Arg is likely to be disruptive. The second mutation, p.Gly80X, occurs in the last exon and so is not likely to trigger nonsense-mediated decay. This residue occurs in the transmembrane region, and the stop codon would truncate the protein and eliminate the transmembrane helix. Gly70 is flanked by Lys69 and Lys72, which probably demarcates the matrix end of the lipid bilayer. Thus, it seems possible that the truncated NDUFB3 would retain some ability to assemble and provide normal interactions on the matrix side.

#### 2.8.2. NDUFB6

The NDUFB6 gene is located on chromosome 9. NDUFB6 is a single-pass membrane protein with its N-terminus found on the matrix side. It is a 15 kDa protein with 127 amino acids, with residues 6–26 forming an alpha helix on the matrix side and residues 56–92 forming an alpha-helix that crosses the membrane. Its initiator methionine is removed during processing. Furthermore, it contains an N-acetylated threonine at residue 2 [58] and a N6-acetylated lysine at residue 24 by analogy with mouse [82]. NDUFB6 contacts NDUFB5, NDUFAB1, and NDUFB9 on the matrix side, core subunit ND5 in the membrane, and supernumerary subunits NDUFB7 and NDUFB10 in the IMS. In a knockout strain of cultured human cells, the loss of NDUFB6 resulted in no assembly of complex I [11].

It has been established that insulin-resistance and type 2 diabetes are associated with reduced NDUFB6 expression [83]. To date, only one polymorphism has been found in NDUFB6 [84]. This polymorphism, rs629566 (A/G or G/G), is located in the promoter region of the NDUFB6 gene. It changes the DNA sequence at position 544 from CA to CG. This polymorphism introduces a fourth potential methylation site in the promoter of the NDUFB6 gene. The introduction of a fourth methylation site resulted in increased DNA methylation at the NDUFB6 gene promoter and decreased NDUFB6 mRNA expression in the muscle of elderly patients. As predicted, elderly patients with the rs629566 G/G genotype had lower NDUFB6 mRNA expression than elderly patients with the A/G genotype. Surprisingly, young patients with the A/G polymorphism had the same level of NDUFB6 mRNA expression as young patients with the wild-type A/A gene. Moreover, young patients with the G/G genotype actually had the highest levels of NDUFB6 mRNA. Thus, it was concluded that age significantly affects NDUFB6 DNA methylation and influences NDUFB6 expression.

Furthermore, it has been suggested that NDUFB6 is a possible tumor suppressor of metastatic clear cell renal cell carcinoma (ccRNC) [85]. In an analysis of 50 primary ccRNC samples, copy number alteration at the 9p24.3–p13.3 region (the chromosomal region in which NDUFB6 gene is located) was found to strongly correlated with poor ccRNC prognosis. Patients missing this chromosomal region had higher cancer recurrence rates, higher metastasis rates, and lower survival rates. It was determined that NDUFB6 expression was downregulated in primary ccRNC samples due to gene copy number loss. More focused experimentation revealed that when NDUFB6-encoded lentiviruses were transduced into a ccRNC cell line, cell proliferation was suppressed. Consequently, it was observed that siRNA knockdown of NDUFB6 led to cell proliferation with the same cell line. The study concluded that loss of region 9p24.1–p13.3 results in NDUFB6 downregulation, which causes cell proliferation in metastatic ccRNC tumors.

#### 2.8.3. NDUFB8

**Figure 17:**
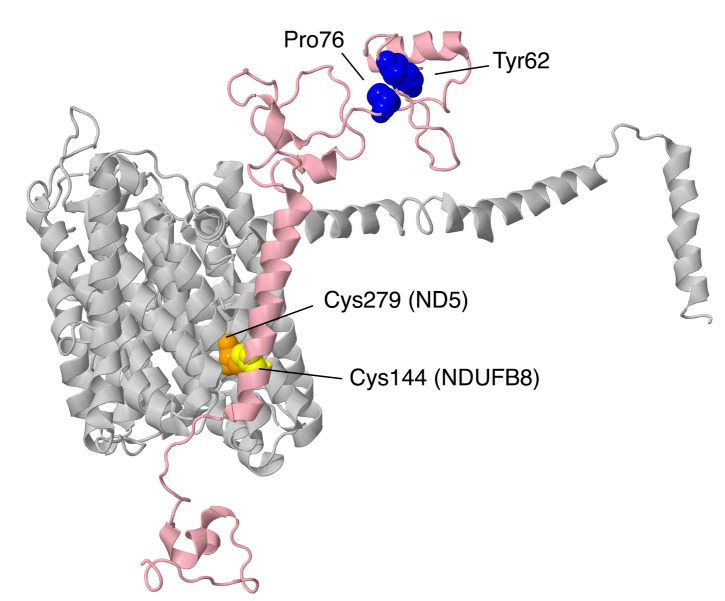
Structural features of NDUFB8 from the ND5-module. The sites of three mutations in NDUFB8 are shown in space-filling. NDUFB8 is colored pink, while the sites of Pro76Gln and Tyr62His are shown in blue on the matrix side, and Cys144Trp is shown in yellow. The Cys279 of ND5 is shown in orange, and it appears to form a disulfide with Cys144 of NDUFB8.

The NDUFB8 gene is located on chromosome 10. The NDUFB8 subunit is initially synthesized with 186 residues and a mass of about 22 kDa, but the first 28 residues serve as a transit peptide and are cleaved off during processing. NDUFB8 is a single-pass protein with the C-terminus in the IMS and the N-terminus in the matrix (see Figure 17). It is located near the end of complex I, and its single transmembrane span lies near ND5. The helix residues (126–151) are not conserved, but the small domains on each side of the helix are much more conserved. On the matrix side NDUFB8 mainly contacts core subunit ND5 and NDUFB4, but it also contacts core subunit ND4 and NDUFB9. On the IMS side, NDUFB8 contacts NDUFB7 and NDUFB10. In a knockout strain of cultured human cells, the loss of NDUFB8 resulted in no assembly of complex I [11].

In 2018, two unrelated compound heterozygous patients with biallelic NDUFB8 mutations were identified to have Leigh-like encephalomyopathy [86]. At three months of age, Patient 1 showed symptoms of failure to thrive, muscle hypotonia, and elevated lactate levels. Patient 2 began to show these symptoms at six months of age. Similar to patients with Leigh syndrome, both patients showed symmetrical basal ganglia and capsula interna lesions. Hypertrophy of the left cardiac ventricle was observed in Patient 1, and he died at 15 months of age. Patient 2 was still alive at six years of age.

Patient 1 harbored a p.Pro76Gln mutation and a p.Cys144Trp mutation. Pro76 is located within a highly conserved region. The residue lies on the matrix side above the lateral helix of core subunit ND5, and it is near core subunit ND4. Cys144 is found within the membrane domain. Though it is weakly conserved, it appears to form a disulfide with Cys279 of chain ND5. Surprisingly, it was discovered that the p.Cys144Trp mutation generated a new exonic splicing silencer (ESS). The Human Splicing Finder algorithm was used to identify the ESS, and Sanger sequencing revealed that exon 4 (Met105–Val156) was skipped in the NDUFB8 transcript, thus confirming the splice defect. Nevertheless, 14% of total cDNA from Patient 1 contained the p.Cys144Trp missense mutation, indicating that while skipping exon 4 is favored, normal splicing also occurs at a lower frequency. Upon the analysis of respiratory chain activity in Patient 1′s muscle tissue and BN gel electrophoresis in a muscle biopsy sample, isolated complex I deficiency was confirmed.

Patient 2 carried a p.Tyr62His mutation and a loss-of-function deletion Glu63Aspfs*35 (c. 189delA). Tyr62 is completely surrounded by mostly conserved residues of NDUFB8 and is located in the same conserved region as Pro76; the two residues are only 4–5 Å distant from each other. Furthermore, the side chain hydroxyl group of Tyr62 forms a hydrogen bond with the side chain and backbone oxygen atoms of Asp74 in NDUFB8. Thus, although His is a fairly conserved substitution for Tyr, His substitution would likely be disruptive at this residue. Complex I deficiency in Patient 2 was observed through the BN gel electrophoresis of muscle homogenate. The complementation of both mutant cell lines was tested using the lentiviral expression of wild-type NDUFB8. Cells from Patient 1 showed normal levels of complex I activity by in-gel assays after BN gel electrophoresis, and normal levels of the NDUFB8 subunit by immunoblotting. Cells from Patient 2 did not grow well enough for such analysis, but both cell lines showed complementation in assays using microrespirometry and flow cytometry.

## 3. Conclusions

This review has discussed 20 of the 30 supernumerary subunits of human complex I. Missense and various other point mutations have been described for 17 of the subunits. One subunit, NDUFV3, has several known amino acids substitutions, but there is no evidence that they are causative for disease. All of the mutations, the diseases they are associated with, and their effects on assembly of complex I are summarized in Table 2. The gene of one subunit NDUFA3 was completely deleted, along with several neighboring genes. This provided evidence for haplosufficiency, since no mitochondrial disease characteristics occurred in those individuals. The gene for another subunit NDUFB6 had a mutation in the promoter, resulting in a change in the expression level.

Among the 17 subunits that were found to have missense or other point mutations, nearly all were homozygous or compound heterozygous and therefore lacked a normal allele. This supports the likely general trend of haplosufficiency. One exception might be in NDUFB11, which is found on the X chromosome. We suggest that mosaicism in female patients due to X-inactivation might explain variable outcomes from heterozygous null mutations. To resolve such questions, it is important to extensively sequence the genome to identify other possible mutations related to the disease state. Another discrepancy was found to occur in NDUFB3, in which one girl with one homozygous mutation died in infancy [78]; meanwhile, several boys with the same mutation, in the hemizygous form, survived into adolescence and adulthood [80]. Deep sequencing might be the answer, again to try to identify other mutations.

Among the missense mutations, many are found at subunit interfaces. Such mutations are predicted to negatively impact assembly, at least local assembly. Others are found at interior sites of the proteins, and it can be predicted that protein folding will be disrupted and lead to a nonfunctional state, an effect similar to that of a gene knockout. The results of the knockouts of each of the supernumerary subunits in cell culture [11] have demonstrated that most are essential for complex I assembly. The same general trend can be seen with missense mutations in the supernumerary subunits. Essential subunits might include those that serve to stabilize the structure of complex I or that might act as chaperones in the assembly process. In recent years, analyses of complex I assembly have increased, but there have not been a great number of mutants analyzed at sufficient depth. The network of interactions among supernumerary subunits, as well as with core subunits, suggests that the instability of complex I might reflect a cooperative assembly and binding of the subunits. The loss of one supernumerary subunit could impact multiple other subunits such that assembly is not completed. Even if complex I is able to assemble, its stability might be marginal, eventually leading to a loss of function.

In conclusion, the increasingly high-resolution structures of complex I from human and other species have helped to interpret the possible roles of the supernumerary subunits and the loss of function due to mutation. Unbiased DNA sequencing will be necessary to find all of the mutations that might exist in various disease states, as illustrated by the mutations discovered in NDUFB11 to be associated with congenital sideroblastic anemia [77]. In this way, other rare diseases might become better understood and treated.

## Figures and Tables

**Table 1:** Clinical missense mutations in supernumerary subunits and their surface contacts. IMS: intermembrane space.

Location	Subunit	Mutated Residues	Primary Contacts
N-module	NDUFA2	p. Lys45Thr p. Glu57Ala p. Asp50Asn + others *	NDUFS1
	NDUFV3	p. Arg26Gln p. Lys56Asn p. Gly103Asp p. Glu276Lys	NDUFV1, NDUFV2
Q-module	NDUFS4	p. Trp114Arg p. Asp119His + others *	NDUFS1, NDUFS3, NDUFS8, NDUFV1, NDUFA6, NDUFA9
	NDUFS6	p. Cys115Tyr + others *	NDUFA9, NDUFA12, NDUFS1, NDUFV2, NDUFS2, NDUFS8
	NDUFA9	p. Arg321Pro p. Arg360Cys	NDUFS3, NDUFS7, NDUFS4, NDUFS6, NDUFA6
ND1- module	NDUFA1	p. Gly8Arg p. Arg37Ser p. Gly32Arg p. Pro19Ser	ND1, NDUFA8
	NDUFA3	Deletion of one allele	ND1, ND3, NDUFS8, NDUFA8, NDUFA13
	NDUFA13	p. Arg57His	ND1, ND6, NDUFS2, NDUFS5, NDUFA3, NDUFA8
LYR proteins	NDUFA6	p. Arg64Pro + others *	NDUFAB1, NDUFS1, NDUFS3, NDUSA9
	NDUFB9	p. Leu64Pro p. Arg47Leu	NDUFB1, NDUFB3, NDUFB4, NDUFB5, NDUFB6
IMS proteins	NDUFS5	p. Pro96Ser	ND2, ND4L, NDUFB5, ND6, NDUFB13
	NDUFA8	p. Arg47Cys p. Glu109Lys p. Arg135Gln	NDUFA13, NDUFA3, NDUFA1
	NDUFB10	Glu79 p. Cys107Ser	NDUFB5, NDUFB6, NDUFB11
ND2-module	NDUFA10	p. Gln142Arg p. Leu294Pro p. Gly99Glu + others	ND2, NDUFS2
	NDUFC2	p. His58Leu + others *	ND2, NDUFC1, NDUFB5, NDUFA8
ND4-module	NDUFA11	p. Ala132Pro p. Thr106Ile (isoform two) + others *	ND2, ND4, ND5, NDUFB5
	NDUFB11	p. Arg134Ser p. Glu219Lys + others	ND4, NDUFB10
ND5-module	NDUFB3	p. Trp22Arg + others	NDUFB9, NDUFAB1, NDUFB2, ND5
	NDUFB6	Promoter mutation	NDUFB5, NDUFAB1, NDUFB9, ND5, NDUFB10, NDUFB7
	NDUFB8	p. Pro76Gln p. Cys144Trp p. Tyr62His + others *	ND5, NDUFB4, ND4, NDUFAB1, NDUFB7, NDUFB9, NDUFB10

* Other mutations include nonsense mutations, splicing mutations, start codon mutations, insertions, and deletions. Mutations are defined by the change in the protein (p.) and the amino acid substitution, Lys45Thr, lysine at position 45 changed to threonine. See text for more details.

**Table 2:** Summary of mutations with associated diseases and effects on assembly.

Gene	Mutation (DNA)	Mutation (Protein)	Diagnosis	Assembly ^1^	Reference
NDUFA2	c.A170 C	p.Glu57Ala	Microcephaly Leukoencephalopathy	NT	[14]
	1. c.134A > C 2. c.225del	1. p.Lys45Thr 2. p.Asn76Metfs*4 ^2^	Leukoencephalopathy	NT	[13]
	c.134 A > C	p.Lys45Thr	Leukoencephalopathy	• Smaller complex	[13]
	c.208 + 5 G > A	mRNA not detected	Leigh syndrome, hypertrophic cardiomyopathy	•	[12]
NDUFV3	1. c.77 G>A 2. POLG: c.32 G > A	1. p.Arg26Gln 2. POLG: p.Gly11Asp	Complex I deficiency	NT	[22]
	1. c.168 A > C 2. POLG: c.2492 A > G 3. ND1 m3946A > G	1. pLys56Asn 2. POLG: p.Tyr831Cys 3. ND1: p.Glu214Lys	Complex I deficiency	NT	[22]
	c.308 G > A	p.Gly103Asp	Complex I deficiency	NT	[22]
	1. c.826 G > A 2. NDUFS8:c.460 G > A	1. p.Glu276Lys 2. NDUFS8: p.Gly154Ser	Complex I deficiency	NT	[22]
	1. c.168 A > C 2. AMACR: c.554 T > C	1. pLys56Asn 2. AMACR: p.Val185Ala	Complex I deficiency	NT	[22]
NDUFS4	AAGTC at 466–470 duplication	frameshift	Leigh-like disease	•	[25]
	c.G289*del	p.Tyr97* ^2^	Leigh-like disease	•	[25]
	c.G44 A	Splicing variants, no protein detected	Leigh-like disease	•	[25]
	1. c.355 G > C 2. c.462delA	1. p.Asp119His 2. p.Lys154Asnfs*34	Leigh syndrome	Smaller size	[28]
	1. c.99-1 G > A .2. c.462delA	1. p.Ser34Ilefs*4 2. p.Lys154Asnfs*34	Leigh syndrome	NT	[22]
	c.340 T > C	p.Trp114Arg	Leigh syndrome	NT	[27]
	c.221delC	p.Thr74Ilefs*17	Leigh syndrome	NT	[22]
NDUFS6	c.344 G > A	p.Cys115Tyr	Neonatal lactic acidemia	NT	[33]
	c.186+2 T > A	Affected splice site, eventually leading to premature termination	Lethal infantile mitochondrial disease	NT	[32]
	1. c.343 C > A 2. c.309 + 5 G > A	1. p.Cys115Arg 2. Loss of exon 3, but some normally spliced	Leigh syndrome	•	[34,35]
NDUFA9	c.962 G > C	p.Arg321Pro	Leigh syndrome	•	[40]
	c.1078 C > T	p.Arg360Cys	Leigh syndrome	••	[87]
NDUFA1	c.22 G > C	p.Gly8Arg	Leigh syndrome	••	[43]
	c.55 C > T	p.Pro19Ser	Leigh syndrome	•	[47]
	c.111 G > C	p.Gly32Arg	Mitochondrial encephalopathy	••	[44,46,88]
	c.251 G > C	p.Arg37Ser	Myoclonic epilepsy	••	[43]
NDUFA13	c.170 G > A	p.Arg57His	Mitochondrial encephalopathy	•	[54]
NDUFA6	1. c.191 G > C 2. c.265 G > T	1. p.Arg64Pro 2. p.Glu89*	Auditory and optic neuropathy	• Lacking N- and Q- modules	[57]
	c.331_332del	p.Glu111Serfs*35	Mitochondrial-related infantile death	• Lacking N- and Q- modules	[57]
	c.3 G > A	p.Met1Ile	Brain disorder	• Lacking N- and Q- modules	[57]
	1. c.309del 2. c.355del	1. p.Met104Cysfs*35 2. p.Leu119Tyrfs*20	Leukoencephalopathy	NT	[57]
NDUFB9	c.140 G > T	p.Arg47Leu. (heterozygous)	Complex 1 deficiency	•••	[60]
	c.191 T > C	p.Leu64Pro	Complex 1 deficiency	•	[60]
NDUFS5	1. c.286 C > T 2. NDUFS8:c.4 C > T	1. p.Pro96Ser 2. NDUFS8: p.Arg2Cys	Complex I deficiency	NT	[22]
NDUFA8	1. c.404 G > A 2. C20orf: c.686 T > C	1. p.Arg135Gln 2. C20orf: p.Leu229Pro	Complex I deficiency	NT	[22]
	c.139 C > T	p.Arg47Cys	Microcephaly and epilepsy	•	[62]
	1. c.325 G > A 2. NDUFS2: c.671 C > T	1. p.Glu109Lys, mRNA not found 2. p.Ala224Val	Neonatal hypotonia and epilepsy	NT	[63]
NDUFB10	1. c.319 T > C 2. c.206_207insT	1. p.Cys107Ser 2. p.Glu70X	Fatal lactic acidosis, cardiomyopathy	•	[64]
NDUFA10	1. c.1 A > G 2. c.425 A > G	1. p.Met1Val 2. p.Gln142Arg	Leigh syndrome	•	[68]
	1. c.891 T > C 2. c.383_384insTAA	1. p.Leu294Pro 2. p.Ser218delinslS	Leigh syndrome	NT	[27]
	c.296 G > A	p.Gly99Glu	Leigh syndrome	NT	[69]
NDUFC2	c.346_*7del	p.His116_Arg119delins21	Leigh syndrome	•	[70]
	c.173 A > T	p.His58Leu	Leigh syndrome	•	[70]
NDUFA11	c.99C + 5 G > A	G to A mutation at exon 1-IVS1 splice junction	Encephalocardiomyopathy and fatal infantile lactic acidemia	NT	[72]
	1. c.317 C > T 2. c.394 G > C	1. p.Thr106Ile (isoform two) 2. p.Ala132Pro	Neuromuscular disorder	NT	[73]
NDUFB11	c.324 T > G	p.Tyr108*	Histiocytoid cardiomyopathy, and microphthalmia	NT	[74]
	c. 255 G > A	p.Trp85*	Histiocytoid cardiomyopathy, and microphthalmia	NT	[74]
	c. 262 C > T	p.Arg88*	Histiocytoid cardiomyopathy, and micropthlamia	NT	[75]
	c.402delG	p.Arg134Serfs*2	Microphthalmia and cardiomyopathy	NT	[75]
	c.361 G > A	p.Glu121Lys	Lethal infantile mitochondrial disorder	•	[27]
	c.276_278del	p.F93del	Congenital optic atrophy and myopathy	NT	[77]
NDUFB3	1. c.64 T > C 2. c.208 G > T	1. p.Trp22Arg 2. p.Gly70X	Muscular hypotonia and lactic acidosis	•	[79]
	1. c.64 T > C	p.Trp22Arg	Muscular hypotonia and lactic acidosis	••	[78,89,81]
NDUFB8	1. c.227 C > A 2. c.432 C > G	1. p.Pro76Gln 2. p.Cys114Trp	Leigh-like disease	•	[86]
	1. c.184 C > G 2. c.189delA	1. p.Tyr62His 2. p.Glu63Aspfs*35	Leigh-like disease	••	[86]

^1^ Assembly: ••• normal or near normal; •• intermediate level; • little or no assembly. NT: not tested. When 2 mutations are listed, they are two alleles of the same gene, or if indicated, one is a second gene. See the text for more details. ^2^ p.Asn76Metfs*4 is a frameshift mutation at codon 76 that converts Asn (AAT) to Met (ATG) with a stop codon appearing 4 codons downstream. p.Tyr97* is a nonsense mutation at Tyr76 to TAG.

## Supplementary Materials

The following are available online at https://www.mdpi.com/2075-1729/10/11/296/s1, Table S1: Interactions of Human Complex I Subunits, Video S1: Structure of Human Complex I.

## References

[1] Fassone E., Rahman S. (2012). Complex I Deficiency: Clinical Features, Biochemistry and Molecular Genetics. J. Med. Genet..

[2] Mayr J.A., Haack T.B., Freisinger P., Karall D., Makowski C., Koch J., Feichtinger R.G., Zimmermann F.A., Rolinski B., Ahting U. (2015). Spectrum of Combined Respiratory Chain Defects. J. Inherit. Metab. Dis..

[3] Formosa L.E., Dibley M.G., Stroud D.A., Ryan M.T. (2018). Building a Complex Complex: Assembly of Mitochondrial Respiratory Chain Complex I. Semin. Cell Dev. Biol..

[4] Agip A.-N.A., Blaza J.N., Fedor J.G., Hirst J. (2019). Mammalian Respiratory Complex I through the Lens of Cryo-EM. Annu. Rev. Biophys..

[5] Fiedorczuk K., Sazanov L.A. (2018). Mammalian Mitochondrial Complex I Structure and Disease-Causing Mutations. Trends Cell Biol..

[6] Parey K., Wirth C., Vonck J., Zickermann V. (2020). Respiratory Complex I—Structure, Mechanism and Evolution. Curr. Opin. Struct. Biol..

[7] Wirth C., Brandt U., Hunte C., Zickermann V. (2016). Structure and Function of Mitochondrial Complex I. Biochim. Biophys. Acta.

[8] Elurbe D.M., Huynen M.A. (2016). The Origin of the Supernumerary Subunits and Assembly Factors of Complex I: A Treasure Trove of Pathway Evolution. Biochim. Biophys. Acta.

[9] Gu J., Wu M., Guo R., Yan K., Lei J., Gao N., Yang M. (2016). The Architecture of the Mammalian Respirasome. Nat. Cell Biol..

[10] Ito M., Morino M., Krulwich T.A. (2017). Mrp Antiporters Have Important Roles in Diverse Bacteria and Archaea. Front. Microbiol..

[11] Stroud D.A., Surgenor E.E., Formosa L.E., Reljic B., Frazier A.E., Dibley M.G., Osellame L.D., Stait T., Beilharz T.H., Thorburn D.R. (2016). Accessory Subunits Are Integral for Assembly and Function of Human Mitochondrial Complex I. Nat. Cell Biol..

[12] Hoefs S.J., Dieteren C.E., Distelmaier F., Janssen R.J., Epplen A., Swarts H.G., Forkink M., Rodenburg R.J.T., Nijtmans L.G., Willems P.H. (2008). NDUFA2 Complex I Mutation Leads to Leigh Disease. Am. J. Hum. Genet..

[13] Perrier S., Gauquelin L., Tétreault M., Tran L., Webb N., Srour M., Mitchell J., Brunel-Guitton C., Majewski J., Long V. (2018). Recessive Mutations in NDUFA2 Cause Mitochondrial Leukoencephalopathy. Clin. Genet..

[14] Alagia M., Cappuccio G., Torella A., D’Amico A., Mazio F., Romano A., Fecarotta S., Casari G., Nigro V., TUDP (2020). Cavitating and Tigroid-Like Leukoencephalopathy in a Case of NDUFA2-Related Disorder. JIMD Rep..

[15] Sjöblom T., Jones S., Wood L.D., Parsons D.W., Lin J., Barber T.D., Mandelker D., Leary R.J., Ptak J., Silliman N. (2006). The Consensus Coding Sequences of Human Breast and Colorectal Cancers. Science.

[16] Bridges H.R., Mohammed K., Harbour M.E., Hirst J. (2017). Subunit NDUFV3 is Present in Two Distinct Isoforms in Mammalian Complex I. Biochim. Biophys. Acta.

[17] Dibley M.G., Formosa L.E., Lyu B., Reljic B., McGann D., Muellner-Wong L., Kraus F., Sharpe A.J., Stroud D.A., Ryan M.T. (2020). The Mitochondrial Acyl-carrier Protein Interaction Network Highlights Important Roles for LYRM Family Members in Complex I and Mitoribosome Assembly. Mol. Cell. Proteom..

[18] Guerrero-Castillo S., Cabrera-Orefice A., Huynen M.A., Arnold S. (2017). Identification and Evolutionary Analysis of Tissue-Specific Isoforms of Mitochondrial Complex I Subunit NDUFV3. Biochim. Biophys. Acta.

[19] Dephoure N., Zhou C., Villén J., Beausoleil S.A., Bakalarski C.E., Elledge S.J., Gygi S.P. (2008). A Quantitative Atlas of Mitotic Phosphorylation. Proc. Natl. Acad. Sci. USA.

[20] Liu F., Lössl P., Rabbitts B.M., Balaban R.S., Heck A.J.R. (2018). The Interactome of Intact Mitochondria by Cross-Linking Mass Spectrometry Provides Evidence for Coexisting Respiratory Supercomplexes. Mol. Cell. Proteom..

[21] Sumegi B., Srere P.A. (1984). Complex I Binds Several Mitochondrial NAD-Coupled Dehydrogenases. J. Biol. Chem..

[22] Calvo S.E., Tucker E.J., Compton A.G., Kirby D.M., Crawford G., Burtt N.P., Rivas M., Guiducci C., Bruno D.L., Goldberger O.A. (2010). High-Throughput, Pooled Sequencing Identifies Mutations in NUBPL and FOXRED1 in Human Complex I Deficiency. Nat. Genet..

[23] Zhou H., Di Palma S., Preisinger C., Peng M., Polat A.N., Heck A.J.R., Mohammed S. (2013). Toward a Comprehensive Characterization of a Human Cancer Cell Phosphoproteome. J. Proteome Res..

[24] De Rasmo D., Palmisano G., Scacco S., Technikova-Dobrova Z., Panelli D., Cocco T., Sardanelli A.M., Gnoni A., Micelli L., Trani A. (2010). Phosphorylation Pattern of the NDUFS4 Subunit of Complex I of the Mammalian Respiratory Chain. Mitochondrion.

[25] Scacco S., Petruzzella V., Budde S., Vergari R., Tamborra R., Panelli D., Heuvel L.P.V.D., Smeitink J.A., Papa S. (2003). Pathological Mutations of the Human NDUFS4 Gene of the 18-kDa (AQDQ) Subunit of Complex I Affect the Expression of the Protein and the Assembly and Function of the Complex. J. Biol. Chem..

[26] Petruzzella V., Panelli D., Torraco A., Stella A., Papa S. (2005). Mutations in theNDUFS4 Gene of Mitochondrial Complex I Alter Stability of the Splice Variants. FEBS Lett..

[27] Kohda M., Tokuzawa Y., Kishita Y., Nyuzuki H., Moriyama Y., Mizuno Y., Hirata T., Yatsuka Y., Yamashita-Sugahara Y., Nakachi Y. (2016). A Comprehensive Genomic Analysis Reveals the Genetic Landscape of Mitochondrial Respiratory Chain Complex Deficiencies. PLoS Genet..

[28] Leshinsky-Silver E., Lebre A.-S., Minai L., Saada A., Steffann J., Cohen S., Rötig A., Munnich A., Lev D., Lerman-Sagie T. (2009). NDUFS4 Mutations Cause Leigh Syndrome With Predominant Brainstem Involvement. Mol. Genet. Metab..

[29] Kmita K., Wirth C., Warnau J., Guerrero-Castillo S., Hunte C., Hummer G., Kaila V.R.I., Zwicker K., Brandt U., Zickermann V. (2015). Accessory NUMM (NDUFS6) Subunit Harbors a Zn-Binding Site and Is Essential for Biogenesis of Mitochondrial Complex I. Proc. Natl. Acad. Sci. USA.

[30] Kmita K., Zickermann V. (2013). Accessory Subunits of Mitochondrial Complex I. Biochem. Soc. Trans..

[31] Yip C.-Y., Harbour M.E., Jayawardena K., Fearnley I.M., Sazanov L.A. (2010). Evolution of Respiratory Complex I. J. Biol. Chem..

[32] Kirby D.M., Salemi R., Sugiana C., Ohtake A., Parry L., Bell K.M., Kirk E.P., Boneh A., Taylor R.W., Dahl H.-H.M. (2004). NDUFS6 Mutations Are a Novel Cause of Lethal Neonatal Mitochondrial Complex I Deficiency. J. Clin. Investig..

[33] Spiegel R., Shaag A., Mandel H., Reich D.S., Penyakov M., Hujeirat Y., Saada A., Elpeleg O., Shalev S.A. (2009). Mutated NDUFS6 is the Cause of Fatal Neonatal Lactic Acidemia in Caucasus Jews. Eur. J. Hum. Genet..

[34] Ogawa E., Shimura M., Fushimi T., Tajika M., Ichimoto K., Matsunaga A., Tsuruoka T., Ishige M., Fuchigami T., Yamazaki T. (2017). Clinical Validity of Biochemical and Molecular Analysis in Diagnosing Leigh Syndrome: A Study of 106 Japanese Patients. J. Inherit. Metab. Dis..

[35] Rouzier C., Chaussenot A., Fragaki K., Serre V., Ait-El-Mkadem S., Richelme C., Paquis-Flucklinger V., Bannwarth S. (2019). NDUFS6 related Leigh Syndrome: A Case Report and Review of the Literature. J. Hum. Genet..

[36] Fearnley I.M., Walker J.E. (1992). Conservation of Sequences of Subunits of Mitochondrial Complex I and Their Relationships with Other Proteins. Biochim. Biophys. Acta.

[37] Agip A.-N.A., Blaza J.N., Bridges H.R., Viscomi C., Rawson S., Muench S.P., Hirst J. (2018). Cryo-EM Structures of Complex I From Mouse Heart Mitochondria in Two Biochemically Defined States. Nat. Struct. Mol. Biol..

[38] Kampjut D., Sazanov L.A. (2019). Structure and Mechanism of Mitochondrial Proton-Translocating Transhydrogenase. Nature.

[39] Stroud D.A., Formosa L.E., Wijeyeratne X.W., Nguyen T.N., Ryan M.T. (2012). Gene Knockout Using Transcription Activator-like Effector Nucleases (TALENs) Reveals That Human NDUFA9 Protein Is Essential for Stabilizing the Junction between Membrane and Matrix Arms of Complex I. J. Biol. Chem..

[40] Bosch B.J.V.D., Gerards M., Sluiter W., Stegmann A.P., Jongen E.L.C., Hellebrekers D.M., Oegema R., Lambrichs E.H., Prokisch H., Danhauser K. (2011). Defective NDUFA9 as a Novel Cause of Neonatally Fatal Complex I Disease. J. Med. Genet..

[41] Baertling F., Sánchez-Caballero L., Brand M.V.D., Fung C.-W., Chan S.-S., Wong V.-N., Hellebrekers D., De Coo I., Smeitink J., Rodenburg R. (2018). NDUFA9 Point Mutations Cause a Variable Mitochondrial Complex I Assembly Defect. Clin. Genet..

[42] Chen R., Fearnley I.M., Peak-Chew S.Y., Walker J.E. (2004). The Phosphorylation of Subunits of Complex I from Bovine Heart Mitochondria. J. Biol. Chem..

[43] Fernandez-Moreira D., Ugalde C., Smeets R., Rodenburg R.J.T., Lopez-Laso E., Ruiz-Falco M.L., Briones P., Martin M.A., Smeitink J.A.M., Arenas J. (2007). X-Linked NDUFA1 Gene Mutations Associated With Mitochondrial Encephalomyopathy. Ann. Neurol..

[44] Potluri P., Davila A., Ruiz-Pesini E., Mishmar D., O’Hearn S., Hancock S., Simon M., Scheffler I.E., Wallace D.C., Procaccio V. (2009). A Novel NDUFA1 Mutation Leads to a Progressive Mitochondrial Complex I-Specific Neurodegenerative Disease. Mol. Genet. Metab..

[45] Bindu P.S., Sonam K., Chiplunkar S., Govindaraj P., Nagappa M., Vekhande C.C., Aravinda H.R., Ponmalar J.J., Mahadevan A., Gayathri N. (2018). Mitochondrial Leukoencephalopathies: A Border Zone Between Acquired and Inherited White Matter Disorders in Children?. Mult. Scler. Relat. Disord..

[46] Mayr J.A., Bodamer O., Haack T.B., Zimmermann F.A., Madignier F., Prokisch H., Rauscher C., Koch J., Sperl W. (2011). Heterozygous Mutation in the X Chromosomal NDUFA1 Gene in a Girl With Complex I Deficiency. Mol. Genet. Metab..

[47] Uehara N., Mori M., Tokuzawa Y., Mizuno Y., Tamaru S., Kohda M., Moriyama Y., Nakachi Y., Matoba N., Sakai T. (2014). New MT-ND6 and NDUFA1 Mutations in Mitochondrial Respiratory Chain Disorders. Ann. Clin. Transl. Neurol..

[48] Fearnley I.M., Skehel J.M., Walker J.E. (1994). Electrospray Ionization Mass Spectrometric Analysis of Subunits of NADH: Ubiquinone Oxidoreductase (Complex I) From Bovine Heart Mitochondria. Biochem. Soc. Trans..

[49] Rak M., Rustin P. (2014). Supernumerary Subunits NDUFA3, NDUFA5 and NDUFA12 Are Required for the Formation of the Extramembrane Arm of Human Mitochondrial Complex I. FEBS Lett..

[50] Abu-Safieh L., Vithana E.N., Mantel I., Holder G.E., Pelosini L., Bird A.C., Bhattacharya S.S. (2006). A Large Deletion in the adRP Gene PRPF31: Evidence That Haploinsufficiency Is the Cause of Disease. Mol. Vis..

[51] Rose A.M., Mukhopadhyay R., Webster A.R., Bhattacharya S.S., Waseem N.H. (2011). A 112 kb Deletion in Chromosome 19q13.42 Leads to Retinitis Pigmentosa. Investig. Ophthalmol. Vis. Sci..

[52] Lufei C., Ma J., Huang G., Zhang T., Novotny-Diermayr V., Ong C.T., Cao X. (2003). GRIM-19, a Death-Regulatory Gene Product, Suppresses Stat3 Activity via Functional Interaction. EMBO J..

[53] Huang G., Lu H., Hao A., Ng D.C.H., Ponniah S., Guo K., Lufei C., Zeng Q., Cao X. (2004). GRIM-19, a Cell Death Regulatory Protein, Is Essential for Assembly and Function of Mitochondrial Complex I. Mol. Cell. Biol..

[54] Angebault C., Charif M., Guegen N., Piro-Megy C., De Camaret B.M., Procaccio V., Guichet P.-O., Hebrard M., Manes G., Leboucq N. (2015). Mutation in NDUFA13/GRIM19 Leads to Early Onset Hypotonia, Dyskinesia and Sensorial Deficiencies, and Mitochondrial Complex I Instability. Hum. Mol. Genet..

[55] Maximo V., Botelho T., Capela J.P., Soares P.C., Lima J.A.C., Taveira A.G., Amaro T., Barbosa A.P., Preto A., Harach H.R. (2005). Somatic and Germline Mutation in GRIM-19, a Dual Function Gene Involved in Mitochondrial Metabolism and Cell Death, Is Linked to Mitochondrion-Rich (Hürthle Cell) Tumours of the Thyroid. Br. J. Cancer.

[56] Angerer H., Radermacher M., Mańkowska M., Steger M., Zwicker K., Heide H., Wittig I., Brandt U., Zickermann V. (2014). The LYR Protein Subunit NB4M/NDUFA6 of Mitochondrial Complex I Anchors an Acyl Carrier Protein and Is Essential for Catalytic Activity. Proc. Natl. Acad. Sci. USA.

[57] Alston C.L., Heidler J., Dibley M., Kremer L.S., Taylor L.S., Fratter C., French C.E., Glasgow R.I., Feichtinger R.G., Delon I. (2018). Bi-allelic Mutations in NDUFA6 Establish Its Role in Early-Onset Isolated Mitochondrial Complex I Deficiency. Am. J. Hum. Genet..

[58] Jacome A.S.V., Rabilloud T., Schaeffer-Reiss C., Rompais M., Ayoub D., Lane L., Bairoch A., Van Dorsselaer A., Carapito C. (2015). N- Terminome Analysis of the Human Mitochondrial Proteome. Proteomics.

[59] Daub H., Olsen J.V., Bairlein M., Gnad F., Oppermann F.S., Körner R., Greff Z., Kéri G., Stemmann O., Mann M. (2008). Kinase-Selective Enrichment Enables Quantitative Phosphoproteomics of the Kinome across the Cell Cycle. Mol. Cell.

[60] Haack T.B., Madignier F., Herzer M., Lamantea E., Danhauser K., Invernizzi F., Koch J., Freitag M., Drost R., Hillier I. (2012). Mutation Screening of 75 Candidate Genes in 152 Complex I Deficiency Cases Identifies Pathogenic Variants in 16 Genes Including NDUFB9. J. Med Genet..

[61] Murari A., Thiriveedi V.R., Mohammad F., Vengaldas V., Gorla M., Tammineni P., Krishnamoorthy T., Sepuri N.B.V. (2015). Human Mitochondrial MIA40 (CHCHD4) is a Component of the Fe–S Cluster Export Machinery. Biochem. J..

[62] Yatsuka Y., Kishita Y., Formosa L.E., Shimura M., Nozaki F., Fujii T., Nitta K.R., Ohtake A., Murayama K., Ryan M.T. (2020). A Homozygous Variant in NDUFA8 is Associated with Developmental Delay, Microcephaly, and Epilepsy Due to Mitochondrial Complex I Deficiency. Clin. Genet..

[63] Bugiani M., Invernizzi F., Alberio S., Briem E., Lamantea E., Carrara F., Moroni I., Farina L., Spada M., Donati M. (2004). Clinical and Molecular Findings in Children with Complex I Deficiency. Biochim. Biophys. Acta.

[64] Friederich M.W., Erdogan A.J., Coughlin C.R., Elos M.T., Jiang H., O’Rourke C.P., Lovell M.A., Wartchow E., Gowan K., Chatfield K.C. (2016). Mutations in the Accessory Subunit Ndufb10 Result in Isolated Complex I Deficiency and Illustrate the Critical Role of Intermembrane Space Import for Complex I Holoenzyme Assembly. Hum. Mol. Genet..

[65] Kampjut D., Sazanov L.A. (2020). The Coupling Mechanism of Mammalian Respiratory Complex I. Science.

[66] Morais V.A., Haddad D., Craessaerts K., De Bock P.-J., Swerts J., Vilain S., Aerts L., Overbergh L., Grünewald A., Seibler P. (2014). PINK1 Loss-of-Function Mutations Affect Mitochondrial Complex I Activity via NdufA10 Ubiquinone Uncoupling. Science.

[67] Beinlich F.R.M., Drees C., Piehler J., Busch K.B. (2015). Shuttling of PINK1 between Mitochondrial Microcompartments Resolved by Triple-Color Superresolution Microscopy. ACS Chem. Biol..

[68] Hoefs S.J.G., Van Spronsen F.J., Lenssen E.W.H., Nijtmans L.G., Rodenburg R.J.T., Smeitink J.A.M., Heuvel L.V.D. (2010). NDUFA10 Mutations Cause Complex I Deficiency in a Patient with Leigh Disease. Eur. J. Hum. Genet..

[69] Minoia F., Bertamino M., Picco P., Severino M., Rossi A., Fiorillo C., Minetti C., Nesti C., Santorelli F.M., Di Rocco M. (2017). Widening the Heterogeneity of Leigh Syndrome: Clinical, Biochemical, and Neuroradiologic Features in a Patient Harboring a NDUFA10 Mutation. JIMD Rep..

[70] Alahmad A., Nasca A., Heidler J., Thompson K., Oláhová M., Legati A., Lamantea E., Meisterknecht J., Spagnolo M., He L. (2020). Bi-Allelic Pathogenic Variants in NDUFC2 Cause Early-Onset Leigh Syndrome and Stalled Biogenesis of Complex I. EMBO Mol. Med..

[71] Andrews B., Carroll J., Ding S., Fearnley I.M., Walker J.E. (2013). Assembly Factors for the Membrane Arm of Human Complex I. Proc. Natl. Acad. Sci. USA.

[72] Berger I., Hershkovitz E., Shaag A., Edvardson S., Saada A., Elpeleg O. (2008). Mitochondrial Complex I Deficiency Caused by a Deleterious NDUFA11 Mutation. Ann. Neurol..

[73] Peverelli L., Legati A., Lamantea E., Nasca A., Lerario A., Galimberti V., Ghezzi D., Lamperti C. (2019). New Missense Variants of NDUFA11 Associated with Late-Onset Myopathy. Muscle Nerve.

[74] Shehata B.M., Cundiff C.A., Lee K., Sabharwal A., Lalwani M.K., Davis A.K., Agrawal V., Sivasubbu S., Iannucci G.J., Gibson G. (2015). Exome Sequencing of Patients With Histiocytoid Cardiomyopathy Reveals a de Novo NDUFB11 Mutation That Plays a Role in the Pathogenesis of Histiocytoid Cardiomyopathy. Am. J. Med. Genet. Part A.

[75] Van Rahden V.A., Fernandez-Vizarra E., Alawi M., Brand K., Fellmann F., Horn D., Zeviani M., Kutsche K. (2015). Mutations in NDUFB11, Encoding a Complex I Component of the Mitochondrial Respiratory Chain, Cause Microphthalmia with Linear Skin Defects Syndrome. Am. J. Hum. Genet..

[76] Rea G., Homfray T., Till J., Roses-Noguer F., Buchan R.J., Wilkinson S., Wilk A., Walsh R., John S., McKee S. (2017). Histiocytoid Cardiomyopathy and Microphthalmia With Linear Skin Defects Syndrome: Phenotypes Linked by Truncating Variants in NDUFB11. Mol. Case Stud..

[77] Lichtenstein D.A., Crispin A.W., Sendamarai A.K., Campagna D.R., Schmitz-Abe K., Sousa C.M., Kafina M.D., Schmidt P.J., Niemeyer C.M., Porter J. (2016). A Recurring Mutation in the Respiratory Complex 1 Protein NDUFB11 is Responsible for a Novel Form of X-Linked Sideroblastic Anemia. Blood.

[78] Calvo S.E., Compton A.G., Hershman S.G., Lim S.C., Lieber D.S., Tucker E.J., Laskowski A., Garone C., Liu S., Jaffe D.B. (2012). Molecular Diagnosis of Infantile Mitochondrial Disease with Targeted Next-Generation Sequencing. Sci. Transl. Med..

[79] Haack T., Haberberger B., Frisch E.-M., Wieland T., Iuso A., Gorza M., Strecker V., Graf E., Mayr J.A., Herberg U. (2012). Molecular Diagnosis in Mitochondrial Complex I Deficiency Using Exome Sequencing. J. Med. Genet..

[80] Alston C.L., Howard C., Oláhová M., Hardy S.A., He L., Murray P.G., O’Sullivan S., Doherty G., Shield J.P.H., Hargreaves I.P. (2016). A Recurrent Mitochondrial p.Trp22Arg NDUFB3 Variant Causes a Distinctive Facial Appearance, Short Stature and a Mild Biochemical and Clinical Phenotype. J. Med. Genet..

[81] Hakim A., Zhang X., Delisle A., Oral E.A., Dykas D., Drzewiecki K., Assis D.N., Silveira M., Batisti J., Jain D. (2019). Clinical Utility of Genomic Analysis in Adults With Idiopathic Liver Disease. J. Hepatol..

[82] Rardin M.J., Newman J.C., Held J.M., Cusack M.P., Sorensen D.J., Li B., Schilling B., Mooney S.D., Kahn C.R., Verdin E. (2013). Label-Free Quantitative Proteomics of the Lysine Acetylome in Mitochondria Identifies Substrates of SIRT3 in Metabolic Pathways. Proc. Natl. Acad. Sci. USA.

[83] Mootha V.K., Lindgren C.M., Eriksson K.F., Subramanian A., Sihag S., Lehar J., Puigserver P., Carlsson E., Ridderstrale M., Laurila E. (2003). PGC-1alpha-Responsive Genes Involved in Oxidative Phosphorylation Are Coordinately Downregulated in Human Diabetes. Nat. Genet..

[84] Ling C., Poulsen P., Simonsson S., Rönn T., Holmkvist J., Almgren P., Hagert P., Nilsson E., Mabey A.G., Nilsson P. (2007). Genetic and Epigenetic Factors Are Associated With Expression of Respiratory Chain Component NDUFB6 in Human Skeletal Muscle. J. Clin. Investig..

[85] Narimatsu T., Matsuura K., Nakada C., Tsukamoto Y., Hijiya N., Kai T., Inoue T., Uchida T., Nomura T., Chisato N. (2014). Downregulation of NDUFB 6 due to 9p24.1-p13.3 Loss Is Implicated in Metastatic Clear Cell Renal Cell Carcinoma. Cancer Med..

[86] Piekutowska-Abramczuk D., Assouline Z., Mataković L., Feichtinger R.G., Koňařiková E., Jurkiewicz E., Stawiński P., Gusic M., Koller A., Pollak A. (2018). NDUFB8 Mutations Cause Mitochondrial Complex I Deficiency in Individuals with Leigh-like Encephalomyopathy. Am. J. Hum. Genet..

[87] Baertling F., Sánchez-Caballero L., Timal S., Brand M.A.V.D., Ngu L.H., Distelmaier F., Rodenburg R.J., Nijtmans L. (2017). Mutations in Mitochondrial Complex I Assembly Factor NDUFAF3 Cause Leigh Syndrome. Mol. Genet. Metab..

[88] Bindu P.S., Sonam K., Govindaraj P., Govindaraju C., Chiplunkar S., Nagappa M., Kumar R., Vekhande C.C., Arvinda H.R., Narayanappa G. (2018). Outcome of Epilepsy in Patients With Mitochondrial Disorders: Phenotype Genotype and Magnetic Resonance Imaging Correlations. Clin. Neurol. Neurosurg..

[89] Alston C.L., Compton A.G., Formosa L.E., Strecker V., Oláhová M., Haack T.B., Smet J., Stouffs K., Diakumis P., Ciara E. (2016). Biallelic Mutations in TMEM126B Cause Severe Complex I Deficiency with a Variable Clinical Phenotype. Am. J. Hum. Genet..

